# MicroRNA Expression Differences in Human Hematopoietic Cell Lineages Enable Regulated Transgene Expression

**DOI:** 10.1371/journal.pone.0102259

**Published:** 2014-07-16

**Authors:** Raul Teruel-Montoya, Xianguo Kong, Shaji Abraham, Lin Ma, Satya P. Kunapuli, Michael Holinstat, Chad A. Shaw, Steven E. McKenzie, Leonard C. Edelstein, Paul F. Bray

**Affiliations:** 1 Cardeza Foundation for Hematologic Research and Department of Medicine, Thomas Jefferson University, Philadelphia, Pennsylvania, United States of America; 2 Departments of Physiology, Pharmacology and Sol Sherry Thrombosis Research Center, Temple University School of Medicine, Philadelphia, Pennsylvania, United States of America; 3 Departments of Molecular and Human Genetics and Medicine, Baylor College of Medicine, Houston, Texas, United States of America; 4 Department of Statistics, Rice University, Houston, Texas, United States of America; University of Nebraska Medical Center, United States of America

## Abstract

Blood microRNA (miRNA) levels have been associated with and shown to participate in disease pathophysiology. However, the hematopoietic cell of origin of blood miRNAs and the individual blood cell miRNA profiles are poorly understood. We report the miRNA content of highly purified normal hematopoietic cells from the same individuals. Although T-cells, B-cells and granulocytes had the highest miRNA content per cell, erythrocytes contributed more cellular miRNA to the blood, followed by granulocytes and platelets. miRNA profiling revealed different patterns and different expression levels of miRNA specific for each lineage. *miR-30c-5p* was determined to be an appropriate reference normalizer for cross-cell qRT-PCR comparisons. miRNA profiling of 5 hematopoietic cell lines revealed differential expression of *miR-125a-5p*. We demonstrated endogenous levels of *miR-125a-5p* regulate reporter gene expression in Meg-01 and Jurkat cells by (1) constructs containing binding sites for *miR-125a-5p* or (2) over-expressing or inhibiting *miR-125a-5p*. This quantitative analysis of the miRNA profiles of peripheral blood cells identifies the circulating hematopoietic cellular miRNAs, supports the use of miRNA profiles for distinguishing different hematopoietic lineages and suggests that endogenously expressed miRNAs can be exploited to regulate transgene expression in a cell-specific manner.

## Introduction

MicroRNAs (miRNAs) function posttranscriptionally in regulating gene expression by inducing mRNA degradation or translation inhibition. More than 2000 human miRNAs have been identified, which are estimated to regulate most (>60%) coding genes [1], [2]. miRNAs regulate genes involved in virtually all physiologic processes and play a critical role for miRNAs in normal lymphopoiesis [3], myelopoiesis [4], erythropoiesis [5] and megakaryocytopoiesis [6]. Dysregulated miRNA expression and function contribute towards the pathogenesis of numerous hematologic diseases, including *miR-29b* in acute myeloid leukemia [7], *miR-145* and *miR-146a* in the 5q- syndrome [8], [9], *mir-125b-2* in acute megakaryoblastic leukemia [10], *miR-28* in myeloproliferative neoplasms [11] and *miR-155*, *miR-21* and *miR-210* in B-cell lymphomas [12].

Besides their importance in disease pathogenesis, miRNAs are increasingly appreciated as a sensitive class of disease biomarkers [13], [14]. miRNAs are relatively easy to measure and are reproducible over time [15], [16]. miRNAs are remarkably stable to extremes of pH, freezing and thawing, and are much more resistant to RNase than mRNA or ribosomal RNA [16]–[18]. These characteristics most likely contribute to the ability of miRNA levels to predict disease activity and survival [17], [19]. Levels of specific platelet miRNAs discriminate essential thrombocytosis from reactive thrombocytosis [20] and mark platelet hyper-responsiveness [21]. *miR-155* levels in B-cells strongly correlate with response to therapy [22] and levels of *miR-223* and *miR-191* vary with the extent of platelet inhibition by thienopyridines and aspirin [23].

Blood miRNAs circulate within cells, microvessicles, exosomes and bound to high-density lipoproteins or Argonaute protein [24], [25]. This systemic delivery enables cell-to-cell transfer of genetic information [26]–[29] and alteration of gene expression in the recipient cell, as has been shown for T-cells to recipient antigen-presenting cells, platelets to endothelial cells, and gut epithelium to T-cells [30]–[32]. Although endothelial, epithelial and perhaps other cells contribute to the extracellular blood miRNA content, most circulating miRNAs are derived from hematopoietic blood cells [33]. To better understand the role of circulating miRNAs in the molecular pathogenesis of hematologic diseases, it is critical to know the cellular source of the miRNAs. Although miRNAs have been profiled for selected hematopoietic lineages [34]–[38], *absolute* quantification of miRNA levels across multiple blood cell types has not been performed. The goals of our study were to quantify the miRNA contents of normal human platelets, T-lymphocytes, B-lymphocytes, granulocytes and erythrocytes on a per cell and per blood volume basis, to determine whether the expression of individual miRNAs differed by cell type, and to explore the potential for exploiting endogenous miRNA levels to modify exogenous gene expression in a hematopoietic cell-specific manner. We found that nucleated cells had substantially higher miRNA content on a per cell basis, but that the hematopoietic cellular contribution to miRNA content of blood on a volume basis was highest in erythrocytes, followed by granulocytes, platelets, T-cells and B-cells. Identification of miRNAs that were differentially expressed (DE) across hematopoietic cell lines enabled cell-specific regulation of transgene expression.

## Methods

### Subjects and peripheral blood cell purification

Donors were 5 healthy males (age 32 years to 56 years), self-identified as white race/ethnicity (Table S1). The study was approved by the institutional review board of Thomas Jefferson University, and written informed consent was obtained from all subjects in accordance with the Declaration of Helsinki.

### Peripheral blood cell purification

Citrated peripheral blood was collected and fractionated over the Ficoll-Histopaque (Sigma, St. Louis, MO, USA). The platelet rich plasma (PRP) layer was removed and platelets were pelleted and resuspended in Beads Buffer (BB; PBS with 0.5% w/v of bovine serum albumin and 2.5 m Methylenediaminetetraacetic acid final concentration). Leukocytes were removed with MACS Human CD45 microbeads reagents (Miltenyi Biotec, Auburn, CA, USA) [39]. The mononuclear cell layer was recovered, washed and re-suspended in BB for isolation of T-cells and B-cells using human CD3 and human CD19 microbeads (MiltenyiBiotec, Auburn, CA, USA), respectively. The buffy coat atop the red blood cells was removed, washed with PBS, treated with Erythrocyte lysis buffer (Qiagen, Hilden, Germany), pelleted and resuspended in BB. Granulocytes were isolated using MACS Human CD15 microbeads (MiltenyiBiotec). Lastly, erythrocytes were isolated from the lowest Ficoll-Histopaque layer by double immunodepletion of white blood cells and granulocytes using Human CD45 and CD15 microbeads. Cell purity was assessed on a FACScan (Becton Dickinson, Franklin Lakes, NJ, USA) using FlowJo 8.5.3 software (Tree Star Inc., Ashland, OR, USA).

### RNA characterization and quantification in blood cell type and blood volume

Cell counts were determined using Hemavet 950 CBC System (The Americas Drew Scientific Inc., CT, USA) prior to RNA extraction. Total RNA was isolated using Trizol Reagent (Invitrogen, Carlsbad, CA) and analyzed on an Agilent 2100 Bioanalyzer (Agilent, Santa Clara, CA), which separates nucleic acid fragments based on their size [40]. The Agilent RNA 6000 Pico Kit was used to assess quantity and integrity of total RNA, as well as the quantity of small (<150 bp) RNA based on area under the curve determinations using Bioanalyzer software. The Agilent Small RNA Pico kit was used for quantification of miRNA by determining the fraction of small RNA that was less than 40 bp. To estimate the contribution of each hematopoietic cell type to miRNA content in blood volume, “cell number per blood volume” was utilized from CBC count for platelets, granulocytes and erythrocytes; CD3+ T-cell and CD19+ B-cell counts were obtained from published reference values [41].

### miRNA profiling, data normalization, relative abundance and cluster analysis

miRNA profiling was performed using the nCounter human miRNA assay kit v1 and v2 and analyzed with the nCounter analysis system (NanoString Technologies, Seattle, WA, USA) [42] and GeneSpring 12.0 GX software (Agilent Technologies, Santa Clara, CA). A total of 623 probe sets were analyzed for all cell types.

miRNA expression levels were normalized to the geometric mean of the 100 highest expressed miRNAs by nSolver software (NanoString Technologies, Seattle, WA, USA). The probe sets from platelets, T-cells, B-cells, granulocytes and hematological cell lines were filtered by calculating the background threshold, defined as the mean of the negative control probe values plus 2 standard deviations (SD). Because erythrocytes showed anomalously low negative control probe values, in order to not include an excess of low expressed but biologically irrelevant miRNAs in erythrocytes, we set the erythrocyte background expression threshold to be similar to the other 4 cell types. For each sample, the background was subtracted from the previous normalized value. Raw data for primary blood cells and hematologic cell lines has been deposited at Gene Expression Omnibus (GEO) under accession number GSE57679.

Normalized miRNA expression levels were transformed into relative abundance data by dividing each individual miRNA count by the total miRNA counts in each sample. The cell type average was calculated and ranked to determine which miRNAs were most abundant. Exploratory cluster analysis and dendrograma were generated using the Pearson correlation with the pairwise complete-linkage method, and the heatmap was generated using the HeatMap Viewer module (GenePattern software; http://www.broadinstitute.org).

### Reporter gene assays

One, two or four miRNA binding site sequences for *miR-125a-5p* or a scrambled control sequence were engineered into a luciferase 3′UTR using pMIR-REPORT vector (Applied Biosystem, Carlsbad, CA, USA) followed by sub-cloning into the pCDH-MSCV-MCS-EF1-GFP vector (System Biosciences). Two million cells per well of Meg-01, Jurkat, Raji and K562 cells were seeded in 6-well plate and transfected with the reporter constructs. Lipofectamine LTX (Invitrogen, Carlsbad, CA, USA) was used to transfect Meg01 Jurkat and K562 cells; Raji cells were transfected using Nucleofector Technology (Lonza AG, Basel, Switzerland). Luciferase assays were performed 24 h post-transfection with the Luciferase assay System (Promega, Madison, WI, USA) using Fluostar OPTIMA (BMG Labtech). The GFP intensity was quantified in a Fluostar OPTIMA (BMG Labtech). Firefly luciferase activity was normalized to GFP intensity.

### Statistical analysis

miRNAs DE among blood cells were identified by comparing normalized miRNA levels in each cell type with the other 4 cell types using the Welch ANOVA and post-hoc test (GeneSpring software, Agilent, Santa Clara, CA, USA). Statistical significance was determined by the Benjamini-Hochberg (BH) correction for multiple testing at *q-value* <0.05, with Tukey post-hoc analysis when the miRNAs were present in all 5 cell types and the Student–Newman–Keuls post-hoc test when a miRNA was absent in one or more cell type. Both NormFinder and Coefficient of Variation methods were used to identify the optimal miRNA for normalization across the five cell types [43]. *T*-tests and Spearman correlations were performed using GraphPad Prism (GraphPad Software, Inc., La Jolla, CA, USA). Data are presented as mean ± SD and p<0.05 was considered statistically significant.

## Results

### Contribution of peripheral blood cell types to the miRNA content of whole blood

**Figure 1:**
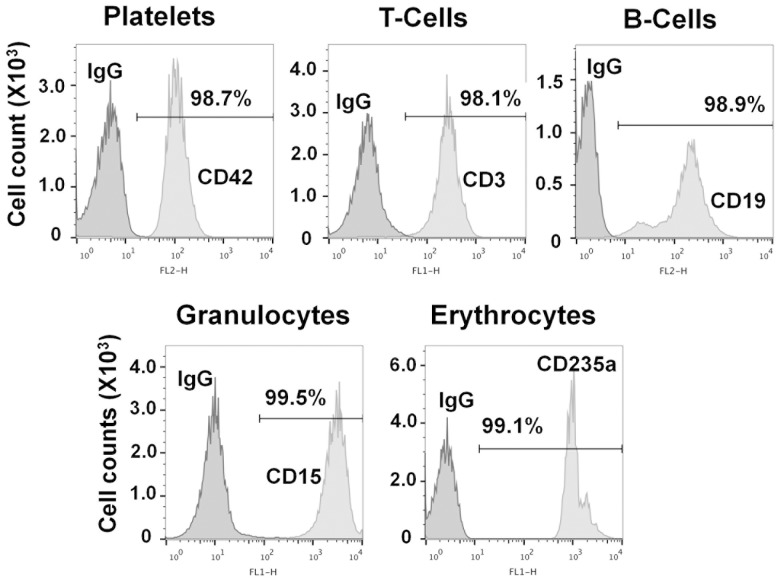
Purity of platelets, granulocytes, T-cells, B-cells and erythrocytes. Cell purity is presented as a histogram of flow cytometric data generated with antibodies CD42, CD3, CD19, CD15 and CD235a specific for platelets, T-cells, B-cells, granulocytes, and erythrocytes, respectively. X-axis is log fluorescence and Y-axis is cell count in ×10^3^ scale.

We determined RNA estimates for each hematopoietic cell type in 3 stages: first, total RNA per cell type; second, miRNA per cell type; and third, cell miRNA per blood volume. We isolated platelets, T-cells, B-cells, granulocytes and erythrocytes from whole blood by density centrifugation and immune-selection from 5 healthy donors. The purity of each cell preparation was greater than 98% (Figure 1).

#### Total RNA per cell type

**Figure 2:**
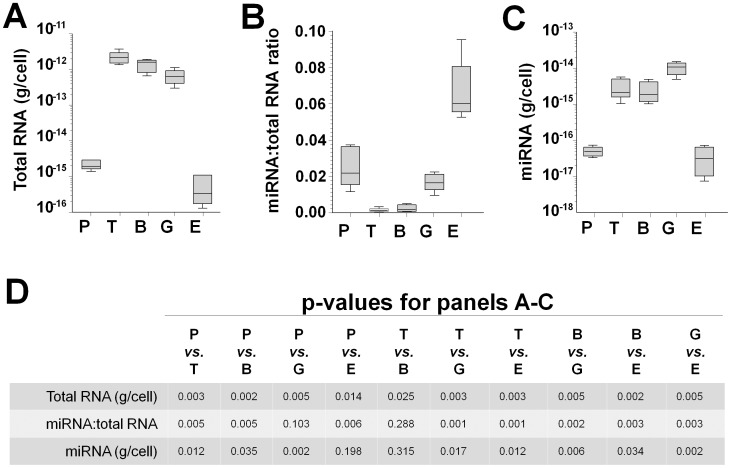
Quantification of total RNA and miRNA in platelets, T-Cells, B-Cells, granulocytes and erythrocytes. (A) Average yield of total RNA from each cell type. (B) Average of miRNA fraction in the total RNA from each cell type. (C) Average miRNA content of each cell type. In (A–C) the box represents the 25th to 75th percentiles, the line in the box is the median and the whiskers represent minimum and maximum values. (E) Summary of comparisons across cell types in panels A–C (one tail t-test). P, platelets; T, T-cells; B, B-cells; G, granulocytes; E, erythrocytes. N = 5 for each of the 5 cell types.

Total RNA was extracted and quantified from 25 cell preparations (5 cell types from 5 donors). The average total RNA yield on a per cell basis from 5 healthy donors revealed nucleated cells contained approximately 1,000 times more total RNA than platelets or erythrocytes (fold-difference range 323 to 3,646) (Figure 2A). On average, T-cells contained 1.61-fold more total RNA than B cells, while B-cells contained 2.10-fold more total RNA than granulocytes (Table 1, row 1). Platelets contained 3.85-fold more total RNA than erythrocytes.

**Table 1:** Contribution of hematopoietic cell type to total miRNA content per volume of blood.

	Platelet	T-Cell	B-Cell	Granulocyte	Erythrocyte
**Total RNA mass, femtogram/cell**	2.20	2,187.97	1,360.34	646.30	0.57
**miRNA mass, femtogram/cell**	0.05	3.28	3.16	10.23	0.04
**miRNA mass, picogram/µl blood**	16.90	3.55	0.64	50.24	184.0

#### miRNA per cell type

We next size-profiled the RNA samples by electrophoretic mobility (Figure S1) and calculated the average miRNA-to-total RNA ratio for each cell type from all subjects. The highest miRNA:total RNA ratios were in erythrocytes and platelets (Figure 2B); the miRNA:total RNA ratios across all cell types were erythrocytes>platelets>granulocytes>lymphocytes. These ratios, coupled with the average total RNA per cell from Figure 2A enabled us to estimate the average miRNA mass per cell type (Figure 2C and Table 1, row 2). T-cells and B-cells contained similar levels of miRNAs, as did platelets and erythrocytes; granulocytes had significant higher miRNA content than other cell types (p values summarized in Figure 2D). On a per cell basis, T-cells, B-cells and granulocytes contained ∼100-fold more miRNA than platelets and erythrocytes.

#### miRNA per blood volume

Lastly, we estimated the contribution of each hematopoietic cell type to the miRNA content of blood volume using cell miRNA content and cell number per blood volume. Notably, erythrocytes contributed the most miRNA to the blood, followed by granulocytes and platelets (Table 1, row 3).

### Peripheral blood miRNA profiles reveal both unique and common expression patterns

**Figure 3:**
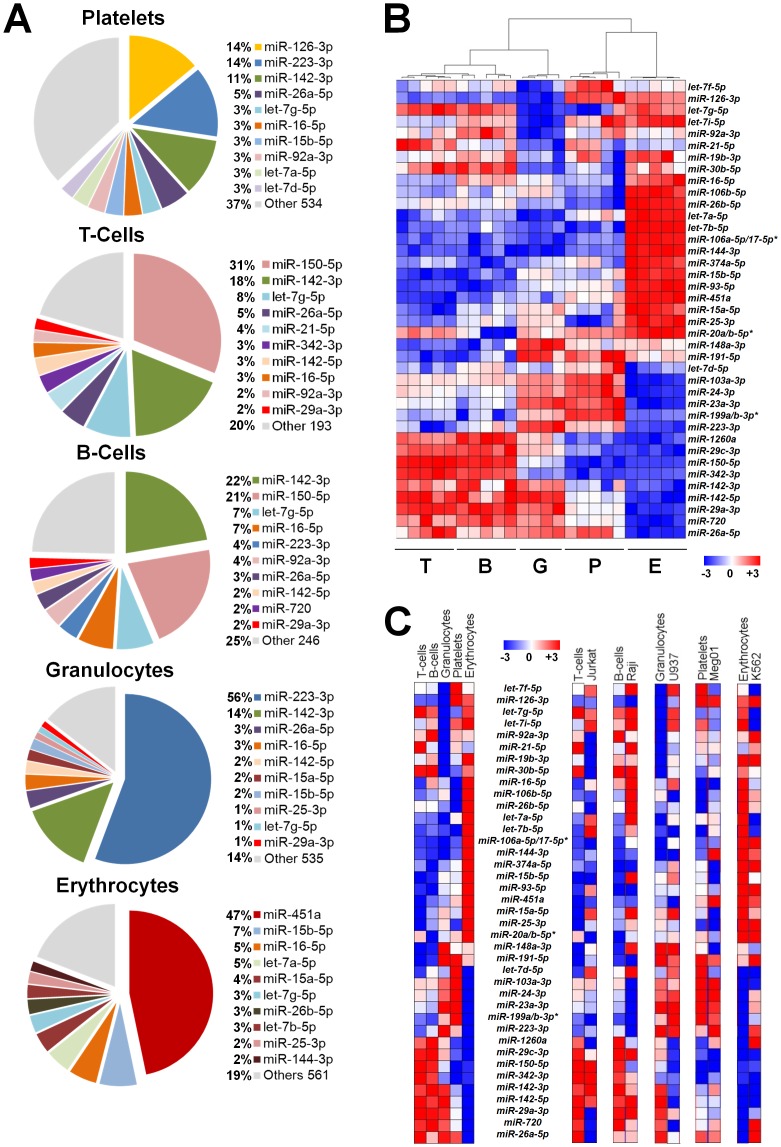
Peripheral blood cells miRNA profiles. (A) Pie graph representation of the top 10 most abundant miRNAs in each cell type. (B) Unsupervised hierarchical clustering of miRNA expression profiles. The dendrogram was generated using the union of the 20 most abundant miRNAs (39 in total) in each cell type. Data from 24 samples was used in these analyses (one granulocyte sample was not analyzed due to technical issues). (C) The left heatmap is derived from the average of the miRNAs shown in panel B. For ease of comparison, the right set of heatmaps display primary miRNA profiles adjacent to corresponding profiles from transformed cell lines, Jurkat (T-lymphoblastic), Raji (B-lymphoblastic), U937 (monocytic), Meg-01 (megakaryoblastic) and K562 (erythroleukemic) cell lines. Each column in the heatmaps indicates the average log-ratio intensity data. * indicates probes with similar and indistinguishable sequence with the nCounter platform.

Besides estimating the total quantities of all miRNA per blood cell type, we quantified levels of *individual* miRNAs for each cell type. Considering only the miRNAs expressed above background, the average number of expressed miRNAs was 544 for platelets, 203 for T-cells, 256 for B-cells, 545 for granulocytes and 571 for erythrocytes (listed in Table S2). Each cell type displayed a similar ∼5 orders of magnitude dynamic range of miRNA expression (supplemental Figure 2), and relatively few miRNAs accounted for the majority of the total cellular content. Figure 3A illustrates the most abundant miRNAs (63%–86% of the total miRNA content, depending on cell type) and emphasizes differences across hematopoietic lineages.

To consider similarities and differences among the different cell types, we performed an unsupervised hierarchical clustering of the most abundant miRNAs (>90% of the total miRNA content for each cell type) (Figure 3B). The resulting dendrogram and heatmap demonstrated that patterns of miRNA expression differ by cell type. Notably, this unbiased analysis based on miRNA expression matches the cell types shown at the bottom of Figure 3B. The similarities and differences in expression patterns were more readily apparent using the average miRNA content per cell type (Figure 3C, left heatmap). T-cells and B-cells had similar miRNA expression patterns that differed from granulocytes, erythrocytes and platelets, which had unique patterns.

Transformed hematopoietic cell lines are often used as models for primary cells, and we profiled miRNAs from Jurkat, Raji, U937, Meg-01 and K562 cells (listed in Table S3). The patterns of miRNA expression in transformed cell lines showed little or modest correlation with *primary* hematopoietic cells (Table S4A; in Figure 3C, right set of heatmaps, compare *miR-126-3p* in the megakaryocyte/platelet lineage, *miR-142-3p* in the lymphocytic lineage, etc.), whereas the cell line expression patterns showed significant correlations among one another (Table S4B).

### Peripheral blood cell source of individual miRNAs

Since different miRNAs may have different biological effects, it is of interest to know the cellular origin of the most abundant blood cell miRNAs. Using cell counts per volume of blood, total miRNA mass per cell and the percentages of individual miRNAs per cell type, we estimated the contribution of the different peripheral blood hematopoietic cells to *individual* miRNAs in the blood (Table 2). The data indicate that different peripheral blood cells contribute unequally to the individual miRNA content in blood. Of note, erythrocytes are the major source for many, but not all, hematopoietic blood cell miRNAs.

**Table 2:** miRNA repertoire per blood volume unit.

Gene Name	Platelets	T-cells	B-cells	Granulocytes	Erythrocytes
***let-7a-5p***	5%	1%	0%	3%	91%
***let-7b-5p***	1%	0%	0%	1%	98%
***let-7d-5p***	36%	2%	1%	24%	38%
***let-7f-5p***	27%	3%	0%	14%	56%
***let-7g-5p***	7%	4%	1%	8%	80%
***let-7i-5p***	15%	1%	0%	4%	80%
***miR-103a-3p***	48%	2%	0%	47%	2%
***miR-106a-5p/17-5p*** *	0%	0%	0%	0%	99%
***miR-106b-5p***	2%	0%	0%	9%	89%
***miR-1260a***	4%	17%	8%	63%	8%
***miR-126-3p***	43%	0%	0%	0%	56%
***miR-142-3p***	13%	5%	1%	58%	23%
***miR-142-5p***	4%	8%	1%	87%	0%
***miR-144-3p***	8%	0%	0%	2%	90%
***miR-148a-3p***	8%	1%	0%	57%	35%
***miR-150-5p***	0%	84%	10%	5%	1%
***miR-15a-5p***	4%	0%	0%	13%	83%
***miR-15b-5p***	4%	0%	0%	7%	89%
***miR-16-5p***	4%	1%	0%	13%	82%
***miR-191-5p***	19%	1%	0%	36%	44%
***miR-199a/b-3p*** *	89%	0%	0%	10%	1%
***miR-19b-3p***	11%	2%	0%	13%	73%
***miR-20a/b-5p***	8%	1%	0%	9%	81%
***miR-21-5p***	21%	8%	1%	15%	55%
***miR-223-3p***	7%	0%	0%	92%	2%
***miR-23a-3p***	35%	1%	0%	63%	1%
***miR-24-3p***	46%	2%	0%	47%	5%
***miR-25-3p***	4%	1%	0%	13%	82%
***miR-26a-5p***	27%	6%	1%	56%	10%
***miR-26b-5p***	0%	0%	0%	1%	98%
***miR-29a-3p***	8%	10%	2%	80%	0%
***miR-29c-3p***	9%	11%	2%	57%	21%
***miR-30b-5p***	10%	4%	1%	14%	71%
***miR-342-3p***	3%	58%	6%	16%	17%
***miR-374a-5p***	2%	0%	0%	2%	96%
***miR-451a***	0%	0%	0%	0%	99%
***miR-720***	12%	9%	2%	75%	1%
***miR-92a-3p***	18%	3%	1%	11%	67%
***miR-93-5p***	1%	0%	0%	1%	98%

Note: each row totals 100%

* Probes with similar and indistinguishable sequence in this assay.

Among the 623 miRNAs queried, 620 were detected in at least one cell type, 3 miRNAs were not detected in any cell type and 165 miRNAs were detected in all the cell types (Table S5A). Additionally, we arbitrarily defined a low expression threshold (less than 10 miRNA counts) and a high expression threshold (greater than 5,000 miRNA counts) (Figure S2). Using these cutoffs, 1% of all detected miRNAs were expressed at high levels and 60% were expressed at low levels. The numbers of miRNAs detected at low levels were 306 for platelets, 68 for T-cells, 159 for B-cells, 295 for granulocytes and 337 for erythrocytes. The numbers of miRNAs detected at high levels were 23 for platelets, 19 for T-cells, 5 for B-cells, 13 for granulocytes and 29 for erythrocyte. *let-7g-5p, miR-142-3p, miR-16-5p* and *miR-223-3p* were expressed at high levels in all 5 lineages, while*miR-134, miR-517c-3p/519a-3p, miR-518d-3p, miR-520d-5p/518a-5p/527 and miR-562* were expressed at low levels in all 5 lineages (Table S5B).

### Identification of miRNAs differentially expressed (DE) across human blood cells

miRNAs regulate gene expression and since different hematopoietic lineages express different repertoires of genes, it is valuable to understand whether miRNAs are DE across human blood cells. To compare across cell lines, miRNA expression values were normalized, background corrected and analyzed for DE with correction for multiple testing. We identified 93 miRNAs that were DE among the 5 blood cell lineages (*q*-value<0.05 by ANOVA). The DE miRNAs for each cell type are listed in Tables S6A–E. Amongst miRNAs exhibiting high expression in at least two of the hematopoietic cell lineages, we identified *miR-142-5p*, *miR-29a-3p*, *miR-150-5p* and *miR-93-5p* as selectively reduced in one or more primary cell types (Table S7).

**Figure 4:**
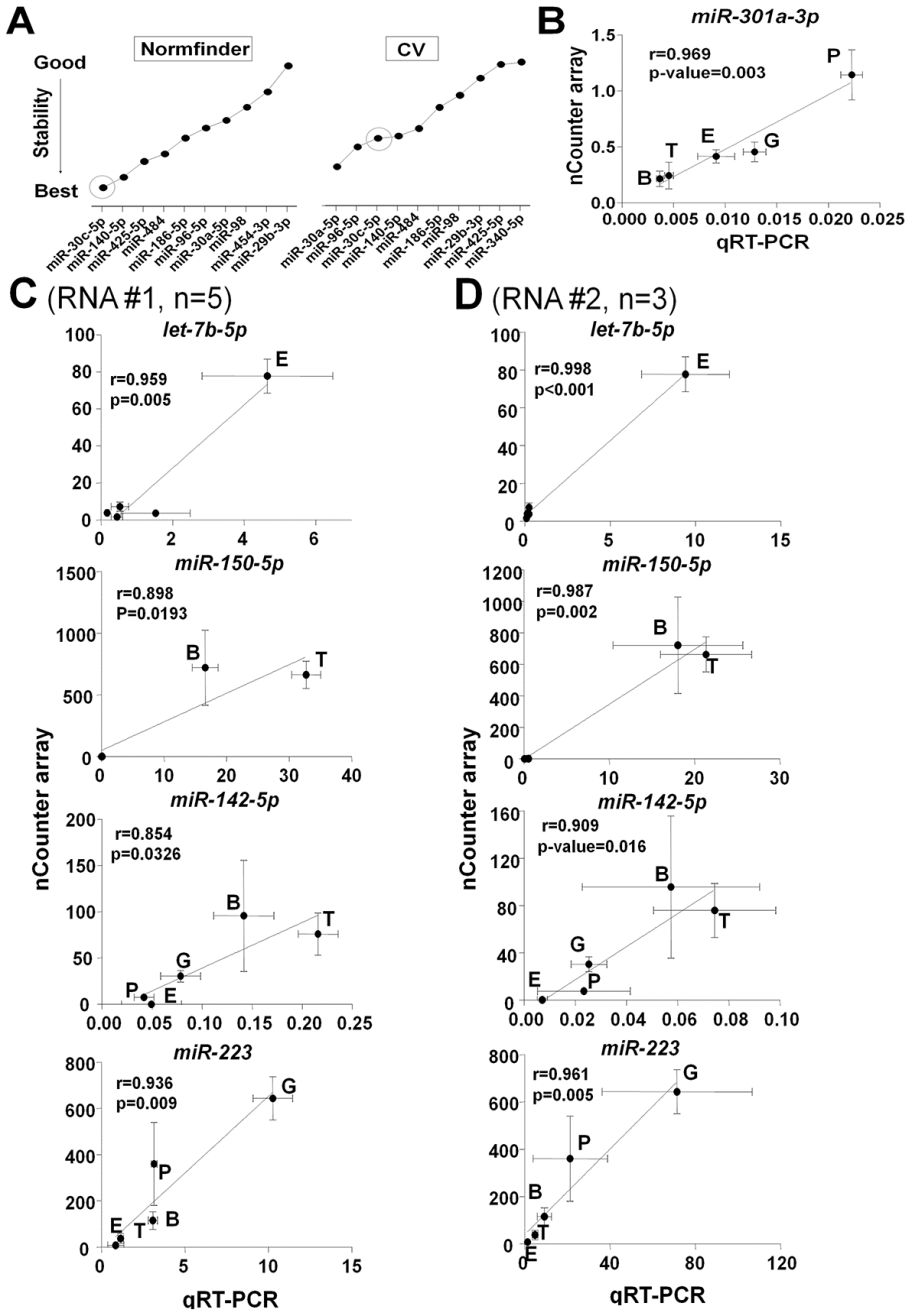
miRNAs DE by cell type. (A) The 10 most stable miRNAs across all cell types are shown for both NormFinder (left) and Coefficient of Variation (CV) methods (right). (B) Validation of NanoString-derived data for *miR-301a-3p*by qRT-PCR data. The X-axis represents the expression level of each miRNA normalized to *miR-30c-3p* using 2^−ΔCt^ method. Each point represents the mean ± SEM of 5 subjects for each cell type. (C,D) Validation of microRNAs DE by cell type. The NanoString-derived data for the indicated miRNAs was validated by qRT-PCR using RNA isolated from the 5 hematopoietic cell types from two different preparations of cells and RNA. qRT-PCR data was normalized and presented as in Figure 4B.P, platelets; T, T-cells; B, B-cells; G, granulocytes; E, erythrocytes.

Validation of DE miRNAs using qRT-PCR requires normalization with an appropriate reference gene, but we were not aware of an established appropriate normalizer for human blood cell miRNAs. As shown in Figure 4A, analysis of our dataset identified several potential normalizers across blood cells. We selected *miR-30c-5p* as a reference normalizer gene because it was moderately expressed and showed minimal variation across all cell types using two different methodologies for assessing variation. The commonly used normalizer, *RNU6B*, was expressed at lower levels and displayed more variability across the 5 blood cell types than *miR-30c-5p* (Table S8). Using *miR-30c-5p* as a normalizer in qRT-PCR, we validated expression levels of *miR-301a-3p* (Figure 4B; r = 0.969, *p*-value = 0.003). Four additional miRNAs were used to validate consistent differential expression of miRNAs across the 5 cell types using two different RNA preparations (Figure 4C–D).

### Development of a model for exploiting endogenous miRNAs to modify exogenous gene expression

**Figure 5:**
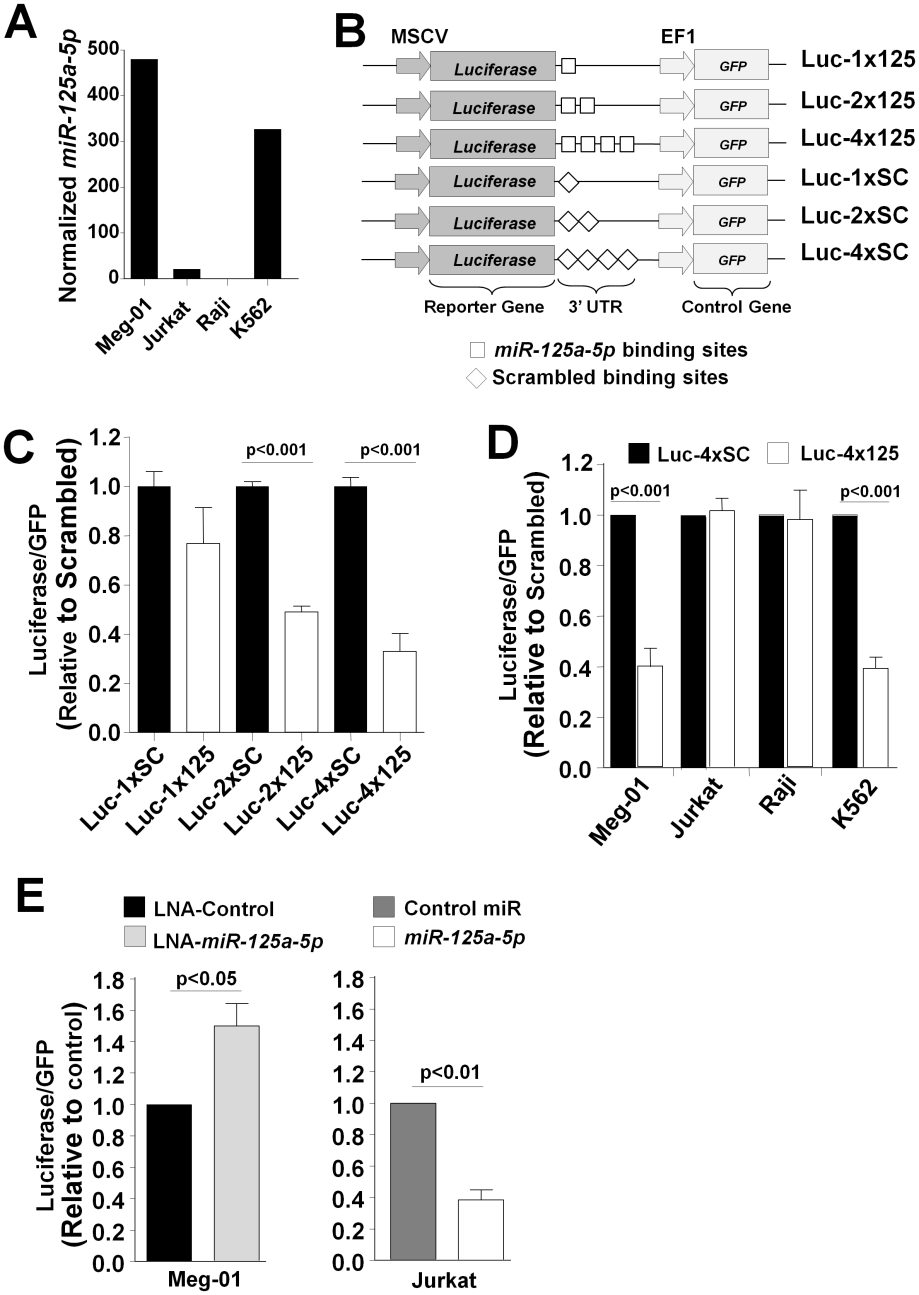
Exploiting endogenous miRNAs to modify exogenous gene expression. (A) Illustration that *miR-125a-5p* was selectively reduced in the lymphocytic cell lines, Jurkat and Raji. (B) Schematic of reporter constructs used to assess effect of endogenous levels of *miR-125a-5p*. Constructs were engineered to contain 1, 2 or 4 *miR-125a-5p* binding sites or scrambled sequence controls. (C) Meg-01 cells were transfected with the indicated constructs. Luciferase repression was enhanced with more *miR-125a-5p* binding sites. (D) Meg-01, Raji, Jurkat and K562 cells were transfected with the 4xSC and 4x125 constructs. Luciferase was quantified and normalized to GFP for transfection efficiency. Data plotted as fold-expression compared to constructs with scrambled sequence. (E) Meg-01 cells were co-transfected with Luc-4x125 construct and control locked nucleic acid (LNA) or LNA that specifically inhibits *miR-125a-5p*. Jurkat cells were co-transfected with Luc-4x125 construct and control pre-miRNA or pre-*miR-125a-5p.* Data in panels C-E are mean ± SD of at least three independent experiments with two replicates each.

To test the hypothesis that endogenous miRNA levels could be exploited to modify transgene expression, we selected *miR-125a-5p*, which was expressed at very low levels in the lymphocytic cell lines, Jurkat and Raji, and high levels in Meg-01 and K562 cells (Figure 5A). We generated a construct containing a luciferase reporter with a 3′UTR containing 1, 2 or 4 tandem *miR-125a-5p* binding sites or scrambled control (Figure 5B). Greater repression of reporter gene expression was observed with the four binding site construct than with the one or two binding site constructs (Figure 5C). Hematological cell lines transfected with only Luc-4x125a or Luc-4xSCR demonstrated lack of transgene repression in Raji and Jurkat cells and ∼60% repression in Meg01 and K562 cells (Figure 5D), consistent with the endogenous expression shown in Figure 5A. To assess whether *miR-125a-5p* directly alters reporter gene repression, we co-transfected Luc-4x125a with an inhibitor of *miR-125a-5p* and observed enhanced expression in Meg-01 cells, whereas over-expression of *miR-125a-5p* repressed reporter gene expression in Jurkat cells (Figure 5E).

## Discussion

Circulating blood miRNAs systemically regulate gene expression and are emerging as important disease biomarkers. We report an unbiased, genome-wide profiling and cross-lineage comparisons of 623 miRNAs from highly purified normal primary human blood platelets, T-cells, B-cells, granulocytes and erythrocytes. The major findings were (1) miRNA profiles differ by hematopoietic lineage, (2) the miRNA content of nucleated cells is approximately 100-fold higher than non-nucleated cells, but erythrocytes contribute the most miRNA mass on a per volume basis (rank order is erythrocyte>granulocyte>platelet>T-cell>B-cell), and (3) differential cell miRNA content can be exploited to regulate exogenous gene expression. We also provide more precise estimates of blood cell RNAs than have been previously reported, and identify appropriate miRNAs for normalization when comparing miRNA measures across hematopoietic cell types. These findings provide a potential refinement for hematopoietic lineage classification, a framework for designing and interpreting miRNA-disease association studies and opportunities to design gene expression vectors that minimize off-target effects.

Estimates of blood cell total RNA content is of interest for optimal design and interpretation of gene expression and biomarker studies. In addition, assessing lineage-specific gene expression requires isolating RNA from highly purified cells. Most prior estimates of blood cell total RNA or miRNA content used density centrifugation for cell purification [38], [44], [45] or could not make quantitative estimates [46]. Unfortunately, density centrifugation alone results in substantial leukocyte contamination of platelet and erythrocyte preparations [47], compromising estimates of the RNA content of non-nucleated cells because of the higher RNA content of nucleated cells. We used density centrifugation followed by immunoselection with cell-specific markers to isolate highly purified populations, an approach considered state-of-the-art for RNA expression analyses [37], [48], [49]. The purification procedure used in the current report yields less than 1 leukocyte per 5 million platelets [21]. In addition, by using the numbers of cells from which RNA was extracted, we were able to make quantitative estimates of RNA per cell. We found the total RNA mass per leukocyte (0.646–2.188 picogram) and per erythrocyte (0.60 femtogram) were similar to other reports [37], [45]. We estimated a total RNA content of 2.20 femtograms per platelet. Prior estimates of platelet RNA content were based on an uncertain number of platelets derived from density centrifugation of buffy coats [44]. Taken together, we conclude that platelets have a slightly higher total RNA content than erythrocytes, but that leukocytes have approximately 1,000 times more RNA than platelets or erythrocytes.

We also estimated absolute miRNA quantities per hematopoietic cell and per blood volume. As expected, T-cells, B-cells and granulocytes had higher miRNA contents than platelets and erythrocytes, most likely because of the greater size and ongoing transcription in leukocytes. However, compared to nucleated cells, platelets and erythrocytes had a higher fraction of miRNA (Figure 2B). Because platelets and erythrocytes have no new RNA synthesis, this difference may simply reflect greater stability of miRNA compared to larger RNAs [18]. There is no reason to expect that platelets and erythrocytes endocytose miRNA to a greater extent than do T-cells, B-cells and granulocytes. One could speculate that circulating non-nucleated cells have evolved to require alternate means of regulating protein translation to maintain viability during their 10 day (platelets) or 120 day (erythrocytes) lifespans. Lymphocytes contain abundant miRNAs and the low miRNA:total RNA ratio (Figure 2B) is likely due to the high content of total RNA.

Although granulocytes, T-cells and B-cells had a much greater abundance of miRNA than platelets or erythrocytes on a per cell basis, erythrocytes, granulocytes and platelets contribute the most miRNA to the content of blood (contribution to blood volume erythrocyte>granulocyte>platelet>T-cell>B-cell). The greater contribution of erythrocytes and platelets to blood volume reflects the considerably higher numbers of these cells in blood compared to leukocytes. Considering the relative abundance of microvessicles originating from these cells [50], erythrocytes, granulocytes and platelets have the potential to have the greatest effect on the systemic effect of miRNA delivery.

Numerous miRNAs were identified as DE across cell types. Although we used qRT-PCR to validate selected miRNA expression levels, we cannot exclude platform-specific miRNA differences that might affect our results. However, such biases would not be expected to be cell-specific, and should not affect our findings of miRNAs DE by cell type. The only hematopoietic cell miRNA profiling study of a large sample size is the Platelet RNA And eXpression-1 (PRAX1) [49]. PRAX1 profiled highly purified platelets and included a heterogeneous population of 154 healthy subjects. Our platelet miRNA profiles showed a very high correlation (p = 5.92×10^-19^) with PRAX1 (Figure S3). Larger studies are needed to address miRNA cell-type dependency and the effects of disease or other demographic variables. Such genome-wide screens require validation, and we determined *miR-30c-5p* to be an ideal internal normalizer for qRT-PCR validation. Notably, *miR-30c-5p* was superior to the commonly used normalizer *RNU6B*, and we would discourage the use of the latter for normalization purposes. Individual miRNAs that were DE by cell type may be useful for identifying the cell of origin of biomarkers or microvessicles and for offering a framework for understanding pathophysiology. In addition, patterns of miRNA expression were highly correlated with cell lineage defined by surface antigens (Figure 3B), consistent with work using mRNA profiles from the Orkin laboratory [51]. Future studies are needed to evaluate miRNA profiles as markers of hematologic disease activity and response to treatment, and to assess whether these DE miRNAs are involved in lineage differentiation.

Several cell-preferentially expressed miRNAs are worth noting. Nearly half the total erythrocyte miRNA content was represented by *miR-451a* (Figure 3A), a finding consistent with its established critical function in erythroid differentiation [52]–[54] and in erythrocyte susceptibility to oxidative stress via*miR-451a*-induced repression of 14-3-3ξ [52], [54]. Similarly, we observed high levels of *miR-150* in both T-cell and B-cells, consistent with the role of this miRNA in lymphoid cell differentiation via its regulation of the c-Myb transcription factor [55]. Older literature refers to *miR-223* as myeloid-specific, but the high level we observed in platelets is consistent with other reports [56], [57], and high levels were also found in Meg-01 cells that display megakaryocytic properties. We found *miR-223* to be the most abundant granulocyte miRNA, consistent with another report using peripheral blood [58] and with the increased expression of *miR-223* that occurs during granulocyte differentiation [59], [60]. It is well-accepted that *miR-223* regulates granulocyte differentiation and function, although the exact molecular mechanism appears complex since ectopic expression of *miR-223* in leukemic cells enhanced myeloid differentiation [59], whereas deletion of *miR-223* in a murine model supported a negative regulatory effect on granulocyte differentiation [60].

Even when tissue-preferential promoters are used to direct transgene expression, off-target and deleterious effects have been observed [61], [62]. The tissue-preferential expression of miRNAs has been exploited to prevent off-target effects in gene therapy studies of mouse models of hemophilia [63]. Brown et al. demonstrated that *mir-142-3p* effectively suppressed transgene expression in hematopoietic lineages in mice, whereas expression was maintained in non-hematopoietic cells [64]. This data is consistent with the identification of *mir-142-3p* as one of 5 highly expressed miRNAs among human peripheral blood cells in our current study. Such a gene therapy approach for hematologic diseases requires knowledge of miRNA levels in different hematopoietic lineages. In the current study, we provide potential miRNAs that could be used to restrict transgene expression to a particular blood cell type (Tables S6 and S7), and demonstrated reporter gene expression could be regulated by endogenous levels of *miR-125a-5p*; high levels of *miR-125a-5p* suppressed expression in a megakaryocytic cell line (Meg-01), whereas low levels of *miR-125a-5p* permitted expression in the lymphocytic-like cell lines (Raji and Jurkat). Unfortunately, the levels of miRNAs identified in primary cells did not correlate well with those detected in the different hematopoietic cell lines (Table S4). Thus, we cannot extrapolate that *miR-125a-5p* would be a useful target for restricting transgene expression in primary cells. But for the purpose of testing the hypothesis of exploiting endogenous levels, our data using this miRNA established the potential for developing gene therapy vectors that exploit *hematopoietic lineage-preferential* miRNA expression.

In summary, we have quantified the total RNA and miRNA contents of normal blood hematopoietic cells and identified miRNAs that are DE in a cell-preferential manner. These data can be utilized as a basis for interpretation of miRNA-disease association studies. For example, if a particular miRNA is elevated in acute myelogenous leukemia (AML), but absent or very low in normal granulocytes (our data), this would suggest this miRNA may participate in the pathogenesis of AML. Knowledge of miRNAs DE by blood cell type is also relevant for understanding the systemic effects of blood cell delivered miRNAs. Since all hematopoietic blood cells release microvessicles upon activation, knowledge of these DE miRNAs is expected to be helpful in understanding systemic effects in response to inflammatory and thrombotic stimuli. Lastly, the demonstration that endogenous miRNA levels can be utilized to regulate transgene expression in hematopoietic cell lines suggests an *in vitro* approach for improving the assessment of gene effects in heterogeneous populations of cultured cells. Considerably more work would be needed to evaluate the value of altering expression vector design for gene therapy of hematological diseases.

## Supporting Information

Figure S1 **Characterization of the small RNA quantities in the total RNA**. **(A–E)** Representative total RNA integrity profiles for each of 5 cell lines using a total eukaryote RNA chip in the Agilent 2100 Bioanalyzer. **(F)** Mean in percentages of small RNA in the total RNA. The fraction of small RNA in the total RNA was calculated from the area under the curve method using image J software (Agilent 2100 Bioanalyzer). The box represents the 25th to 75th percentiles, the line in the box is the median and the whiskers represent minimum and maximum values. Data from 5 subjects (n = 25 samples) was used in these analyses.
(TIF)

Figure S2 **Human peripheral blood cell miRNA expression distribution.** All miRNAs expressed above background are represented on this plot. The individual miRNAs are arbitrarily ordered on x-axis from lowest to highest expressed, and for clarity are represented as a line for each cell type (a bar graph would be visually difficult to present in a single plot). Y-axis is miRNA expression levels in log10 scale and demonstrates a similar ∼5 orders of magnitude dynamic range of miRNA expression for all cell types. Horizontal dashed lines indicate arbitrary high and low expression thresholds.
(TIF)

Figure S3 **Platelet miRNA expression correlations.** The 50 highest expressed platelet miRNAs were considered from the current study and the PRAX1 study (Edelstein et al. Nat Med 2013). (A) Venn-diagram showing 47 of 50 miRNAs were shared between both studies. (B) Pearson correlation between miRNAs in both studies. Points represent the mean of 5 subjects in the current study and the mean of 154 subjects in the PRAX1 study.
(TIF)

Table S1 **Demographic table.**
(DOCX)

Table S2 **miRNA profile in peripheral blood cells.**
(XLS)

Table S3 **miRNA profile in hematopoietic cell lines.**
(XLS)

Table S4 A: Correlations between hematopoietic cell line and primary cell miRNA profiles. B: Correlations between hematopoietic cell line miRNA profiles.
(DOCX)

Table S5 A: Number of miRNA non-detected and detected. B: Number of miRNAs with low or high expression levels.
(DOCX)

Table S6 A: miRNAs DE in platelets compared with all other cell types. B: miRNAs DE in T-cells compared with all other cell types. C: miRNAs DE in B-cells compared with all other cell types. D: miRNAs DE in granulocytes compared with all other cell types. E: miRNAs DE in erythrocytes compared with all other cell types.
(DOCX)

Table S7 **Selectively reduced miRNAs amongst abundantly expressed miRNAs.**
(DOCX)

Table S8 ***miR-30c-5p*** **validation.**
(DOCX)
