# Supplementary figures and images for: MicroRNA Expression Differences in Human Hematopoietic Cell Lineages Enable Regulated Transgene Expression
Source: PLoS One. 2014 Jul 16;9(7):e102259. doi: 10.1371/journal.pone.0102259 (PMC4100820; doi:10.1371/journal.pone.0102259)

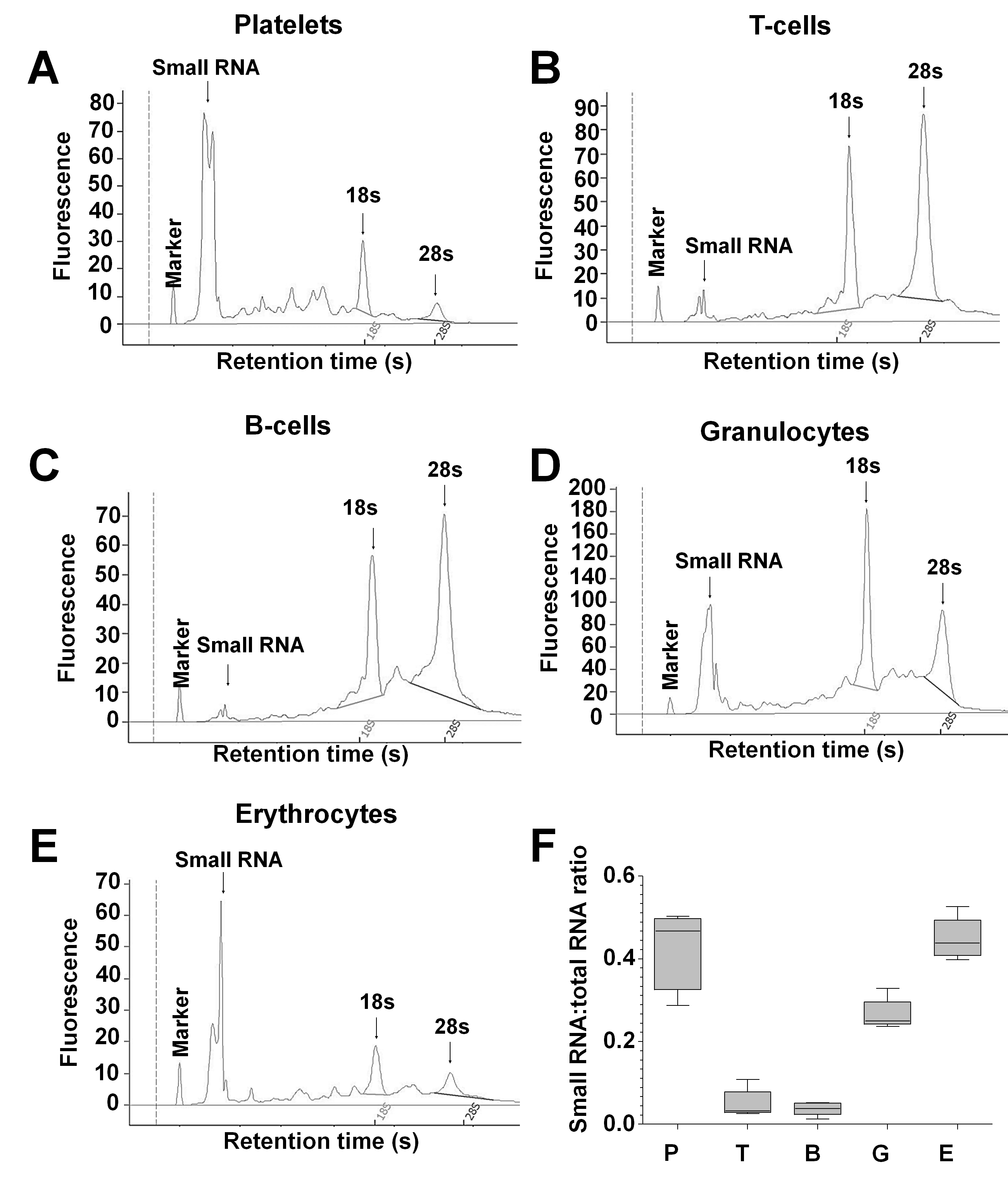

Supplement: Figure S1 — Characterization of the small RNA quantities in the total RNA. (A–E) Representative total RNA integrity profiles for each of 5 cell lines using a total eukaryote RNA chip in the Agilent 2100 Bioanalyzer. (F) Mean in percentages of small RNA in the total RNA. The fraction of small RNA in the total RNA was calculated from the area under the curve method using image J software (Agilent 2100 Bioanalyzer). The box represents the 25th to 75th percentiles, the line in the box is the median and the whiskers represent minimum and maximum values. Data from 5 subjects (n = 25 samples) was used in these analyses. (TIF) [file pone.0102259.s001.tif]

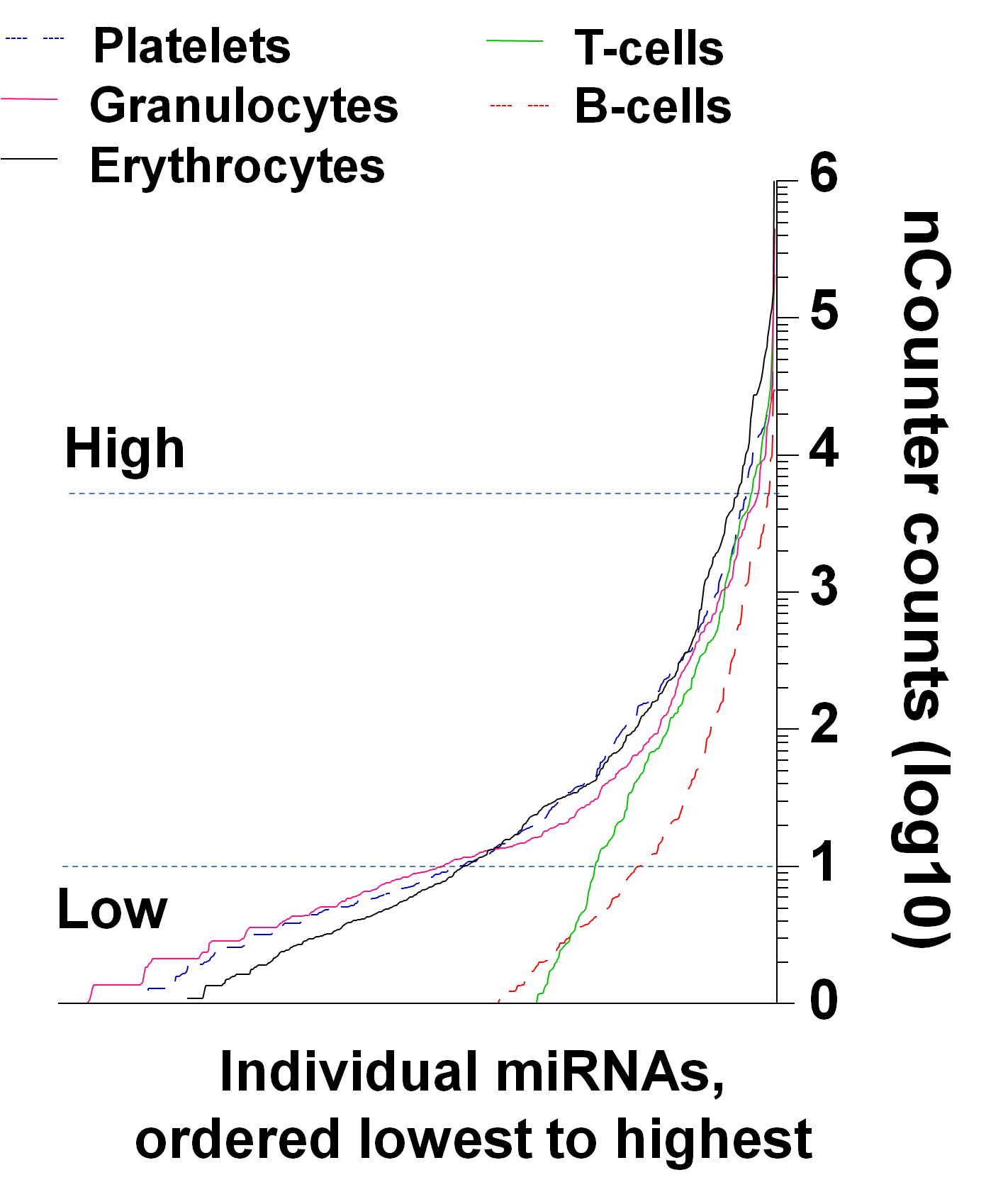

Supplement: Figure S2 — Human peripheral blood cell miRNA expression distribution. All miRNAs expressed above background are represented on this plot. The individual miRNAs are arbitrarily ordered on x-axis from lowest to highest expressed, and for clarity are represented as a line for each cell type (a bar graph would be visually difficult to present in a single plot). Y-axis is miRNA expression levels in log10 scale and demonstrates a similar ∼5 orders of magnitude dynamic range of miRNA expression for all cell types. Horizontal dashed lines indicate arbitrary high and low expression thresholds. (TIF) [file pone.0102259.s002.tif]

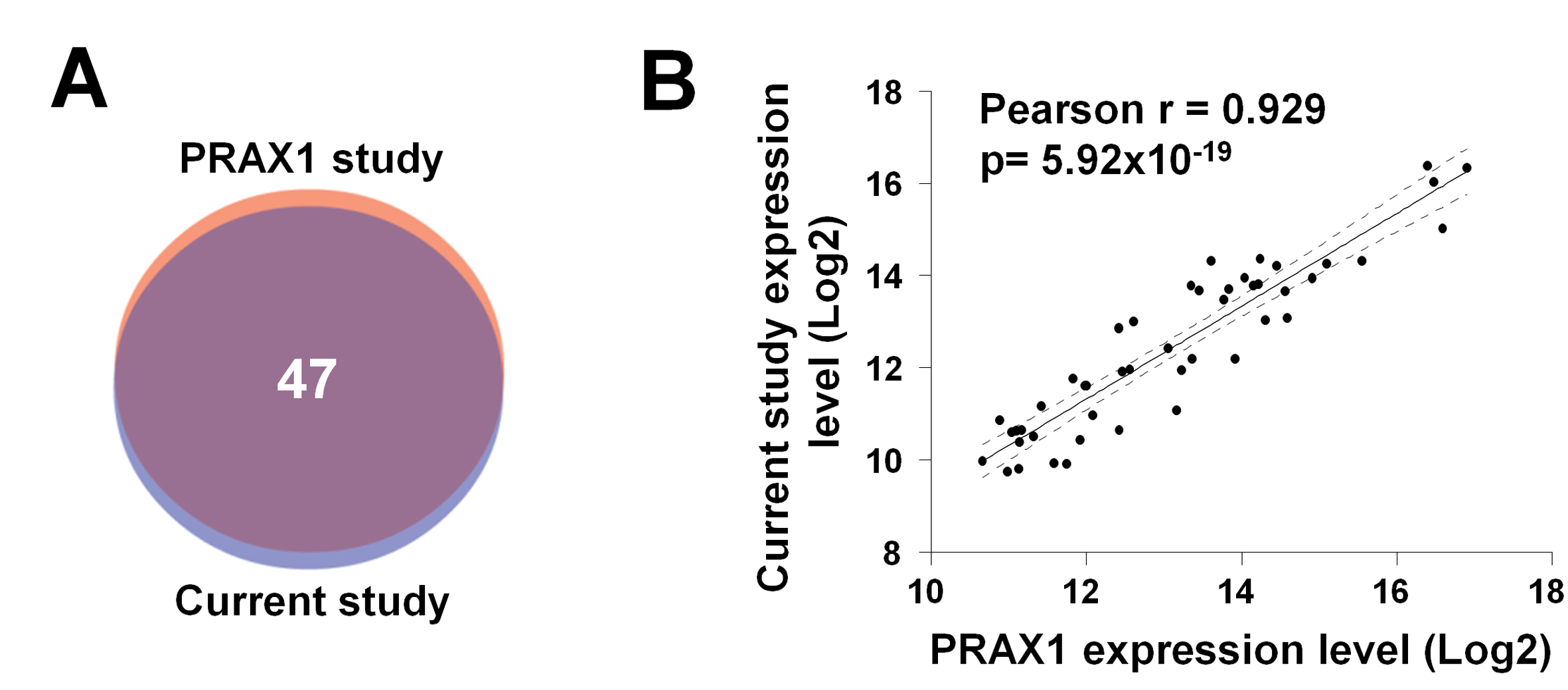

Supplement: Figure S3 — Platelet miRNA expression correlations. The 50 highest expressed platelet miRNAs were considered from the current study and the PRAX1 study (Edelstein et al. Nat Med 2013). (A) Venn-diagram showing 47 of 50 miRNAs were shared between both studies. (B) Pearson correlation between miRNAs in both studies. Points represent the mean of 5 subjects in the current study and the mean of 154 subjects in the PRAX1 study. (TIF) [file pone.0102259.s003.tif]
