# Supplementary material for: MicroRNA Expression Differences in Human Hematopoietic Cell Lineages Enable Regulated Transgene Expression
Source: PLoS One. 2014 Jul 16;9(7):e102259. doi: 10.1371/journal.pone.0102259 (PMC4100820; doi:10.1371/journal.pone.0102259)
Supplement: Table S1 — Demographic table. (DOCX) [file pone.0102259.s004.docx]

**Table S1.Demographic table.**

| **Characteristic** | **Values** |
| --- | --- |
| **Age, mean (range)** | 45.6 (32-56) |
| **Gender, % male** | 100 |
| **Race,% white** | 100 |
| **Smoker,%** | 0 |
| **Hypertension,%** | 0 |
| **Diabetes mellitus, %** | 0 |
| **WBC,x10^3^/µl** | 4.9±0.8* |
| **Hematocrit,%** | 40.2±4.9* |
| **Erythrocytes, x10^6^/µl** | 4.6±0.5* |
| **Hemoglobin, g/dL** | 14.2±1.3* |
| **Platelets, x10^3^/µl** | 339±73* |

*mean±SD.

WBC, white blood cell count
