# Supplementary material for: MicroRNA Expression Differences in Human Hematopoietic Cell Lineages Enable Regulated Transgene Expression
Source: PLoS One. 2014 Jul 16;9(7):e102259. doi: 10.1371/journal.pone.0102259 (PMC4100820; doi:10.1371/journal.pone.0102259)
Supplement: Table S4 — A: Correlations between hematopoietic cell line and primary cell miRNA profiles. B: Correlations between hematopoietic cell line miRNA profiles. (DOCX) [file pone.0102259.s007.docx]

**Table S4 A. Correlations between hematopoietic cell line and primary cell miRNA profiles.**

|  |  | **T-cells** | **Platelets** | **Granulocytes** | **B-cells** | **Erythrocytes** |
| --- | --- | --- | --- | --- | --- | --- |
| **Jurkat** | **# in common top 40** | 14 | 11 | 12 | 13 | 13 |
|  | **Pearson r** | 0.39 | 0.36 | 0.52 | 0.30 | 0.36 |
|  | ***P*-value** | 0.17 | 0.27 | 0.08 | 0.31 | 0.22 |
| **Meg01** | **# in common top 40** | 12 | 8 | 9 | 11 | 10 |
|  | **Pearson r** | 0.51 | 0.31 | 0.57 | 0.40 | 0.16 |
|  | ***P*-value** | 0.09 | 0.45 | 0.11 | 0.22 | 0.65 |
| **K562** | **# in common top 40** | 12 | 10 | 13 | 12 | 12 |
|  | **Pearson r** | 0.60 | 0.20 | 0.66 | 0.45 | 0.24 |
|  | ***P*-value** | 0.04 | 0.58 | 0.01 | 0.14 | 0.46 |
| **Raji** | **# in common top 40** | 17 | 14 | 16 | 16 | 13 |
|  | **Pearson r** | 0.56 | 0.31 | 0.55 | 0.54 | 0.14 |
|  | ***P*-value** | 0.02 | 0.28 | 0.02 | 0.03 | 0.64 |
| **U937** | **# in common top 40** | 16 | 13 | 14 | 15 | 14 |
|  | **Pearson r** | 0.65 | 0.44 | 0.48 | 0.57 | 0.33 |
|  | ***P*-value** | 0.006 | 0.14 | 0.081 | 0.02 | 0.25 |

**Table S4 B. Correlations between hematopoietic cell line miRNA profiles.**

|  |  | Jurkat | U937 | Raji | K562 |
| --- | --- | --- | --- | --- | --- |
| Meg01 | **# in common top 40** | 23 | 26 | 22 | 29 |
|  | **Pearson r** | 0.84 | 0.80 | 0.85 | 0.88 |
|  | ***P*-value** | 6.61E-07 | 7.21E-07 | 6.3E-07 | 4.34E-10 |
| K562 | **# in common top 40** | 26 | 29 | 26 |  |
|  | **Pearson r** | 0.71 | 0.76 | 0.80 |  |
|  | ***P*-value** | 4.08E-05 | 1.53E-06 | 9.42E-07 |  |
| Raji | **# in common top 40** | 31 | 31 |  |  |
|  | **Pearson r** | 0.85 | 0.90 |  |  |
|  | ***P*-value** | 1.54E-09 | 3.23E-12 |  |  |
| U937 | **# in common top 40** | 32 |  |  |  |
|  | **Pearson r** | 0.85 |  |  |  |
|  | ***P*-value** | 4.95E-10 |  |  |  |
