# Supplementary material for: MicroRNA Expression Differences in Human Hematopoietic Cell Lineages Enable Regulated Transgene Expression
Source: PLoS One. 2014 Jul 16;9(7):e102259. doi: 10.1371/journal.pone.0102259 (PMC4100820; doi:10.1371/journal.pone.0102259)
Supplement: Table S5 — A: Number of miRNA non-detected and detected. B: Number of miRNAs with low or high expression levels. (DOCX) [file pone.0102259.s008.docx]

**Table S5 A. Number of miRNAs non-detected and detected.**

|  | Non detected | Detected |
| --- | --- | --- |
| Platelets | 79 | 544 |
| T-cells | 420 | 203 |
| B-cells | 367 | 256 |
| Granulocytes | 78 | 545 |
| Erythrocytes | 52 | 571 |
| All Cells | 3* | 165 |

* *miR-1257, miR-217 and miR-564*

**Table S5 B. Number of miRNAs with low or high expression levels.**

|  | Low | High |
| --- | --- | --- |
| Platelets | 306 | 23 |
| T-cells | 68 | 19 |
| B-cells | 159 | 5 |
| Granulocytes | 295 | 13 |
| Erythrocytes | 337 | 29 |
| All Cells | 4* | 4^†^ |

* *miR-134, miR-517c-3p/519a-3p, miR-518d-3p, miR-520d-5p/518a-5p/527 and miR-562*

^†^*let-7g-5p, miR-142-3p, miR-16-5p and miR-223-3p*
