# Supplementary material for: MicroRNA Expression Differences in Human Hematopoietic Cell Lineages Enable Regulated Transgene Expression
Source: PLoS One. 2014 Jul 16;9(7):e102259. doi: 10.1371/journal.pone.0102259 (PMC4100820; doi:10.1371/journal.pone.0102259)
Supplement: Table S6 — A: miRNAs DE in platelets compared with all other cell types. B: miRNAs DE in T-cells compared with all other cell types. C: miRNAs DE in B-cells compared with all other cell types. D: miRNAs DE in granulocytes compared with all other cell types. E: miRNAs DE in erythrocytes compared with all other cell types. (DOCX) [file pone.0102259.s009.docx]

**Table S6 A. miRNAs DE in platelets compared with all other cell types.**

|  | Counts |  | Counts | Fold(Log2) | Counts | Fold(Log2) | Counts | Fold(Log2) | Counts | Fold(Log2) |
| --- | --- | --- | --- | --- | --- | --- | --- | --- | --- | --- |
| Gene Name | **Platelets** | ***q*-value** | **T-cells** | **T-cells *vs.* Platelets** | **B-cells** | **B-cells *vs.* Platelets** | **Granulo-**  **cytes** | **Gran. *vs.* Platelets** | **Erythro-**  **cytes** | **Eryth. *vs.* Platelets** |
| *let-7a-5p* | 18937 | 1.65E-10 | 8670 | -1.2 | 1796 | -3.4 | 4880 | -1.9 | 105131 | 2.5 |
| *let-7b-5p* | 1168 | 4.26E-10 | 1147 | 0.0 | 304 | -1.9 | 1020 | -0.2 | 56326 | 5.6 |
| *let-7c* | 87 | 1.01E-07 | 132 | 0.6 | 13 | -3.2 | 88 | 0.0 | 4830 | 5.9 |
| *let-7d-5p* | 15925 | 7.84E-05 | 3639 | -1.8 | 1157 | -3.5 | 3962 | -1.7 | 5032 | -1.4 |
| *let-7f-5p* | 13395 | 5.05E-07 | 5421 | -1.3 | 1133 | -3.5 | 2547 | -2.4 | 8348 | -0.6 |
| *let-7g-5p* | 21228 | 4.59E-07 | 55710 | 1.7 | 9844 | -0.9 | 9253 | -0.9 | 73217 | 2.0 |
| *let-7i-5p* | 11471 | 4.59E-07 | 3862 | -1.5 | 1421 | -3.0 | 1045 | -3.4 | 18282 | 0.7 |
| *miR-101-3p* | 567 | 1.78E-07 | 2469 | 2.4 | 458 | -0.2 | 1888 | 2.0 | 226 | -1.1 |
| *miR-103a-3p* | 12976 | 6.66E-09 | 2758 | -2.1 | 573 | -4.4 | 4774 | -1.3 | 142 | -6.5 |
| *miR-106a-5p/17-5p** | 365 | 2.56E-11 | 252 | -0.5 | 50 | -2.9 | 171 | -1.1 | 31312 | 6.4 |
| *miR-106b-5p* | 1607 | 5.56E-08 | 1870 | 0.3 | 538 | -1.6 | 3466 | 1.1 | 27992 | 4.2 |
| *miR-107* | 891 | 8.51E-07 | 91 | -3.6 | 12 | -6.8 | 263 | -1.7 | 2704 | 1.6 |
| *miR-10a-5p* | 109 | 2.61E-02 | 76 | -0.3 | 22 | -2.6 | 42 | -1.8 | 11 | -4.3 |
| *miR-125b-5p* | 162 | 1.14E-03 | 463 | 1.3 | 9 | -5.3 | 53 | -1.6 | 182 | 0.0 |
| *miR-1260a* | 185 | 3.05E-06 | 3816 | 4.4 | 2105 | 3.6 | 1222 | 2.6 | 127 | -0.6 |
| *miR-126-3p* | 85658 | 3.37E-08 | 450 | -7.4 | 85 | -9.9 | 148 | -9.0 | 32963 | -1.2 |
| *miR-128* | 151 | 2.73E-04 | 49 | -1.6 | 15 | -4.2 | 118 | -0.4 | 123 | -0.3 |
| *miR-1283* | 6 | 7.84E-05 | 72 | 3.7 | 124 | 4.3 | 87 | 4.0 | 238 | 5.4 |
| *miR-130b-3p* | 153 | 2.36E-04 | 26 | -2.6 | 4 | -6.0 | 39 | -2.0 | 63 | -1.2 |
| *miR-140-3p* | 21 | 6.86E-07 | 366 | 4.2 | 91 | 2.2 | 72 | 1.9 | 142 | 2.8 |
| *miR-140-5p* | 253 | 8.38E-07 | 429 | 0.8 | 61 | -2.0 | 860 | 1.8 | 475 | 1.0 |
| *miR-142-3p* | 67408 | 2.73E-04 | 119999 | 1.0 | 30119 | -1.2 | 112362 | 0.9 | 35909 | -0.7 |
| *miR-142-5p* | 2167 | 2.12E-07 | 20928 | 3.3 | 3175 | 0.2 | 18903 | 3.2 | 37 | -6.0 |
| *miR-146a-5p* | 3964 | 3.50E-09 | 3614 | -0.2 | 325 | -3.8 | 31 | -7.0 | 76 | -5.7 |
| *miR-146b-5p* | 40 | 4.56E-07 | 2186 | 5.8 | 34 | -1.9 | 18 | -1.1 | 96 | 1.3 |
| *miR-148a-3p* | 1446 | 2.01E-07 | 426 | -1.7 | 101 | -4.0 | 4168 | 1.6 | 2035 | 0.5 |
| *miR-148b-3p* | 3993 | 9.52E-07 | 507 | -2.9 | 138 | -4.9 | 1531 | -1.3 | 1597 | -1.3 |
| *miR-150-5p* | 38 | 2.56E-11 | 207293 | 12.5 | 28880 | 9.7 | 1090 | 4.9 | 90 | 1.4 |
| *miR-1537* | 99 | 4.59E-07 | 122 | 0.5 | 27 | -1.3 | 1687 | 4.7 | 47 | -0.5 |
| *miR-155-5p* | 37 | 3.55E-07 | 711 | 4.3 | 237 | 2.6 | 17 | -1.4 | 97 | 1.4 |
| *miR-15a-5p* | 14117 | 1.78E-07 | 4075 | -1.6 | 2009 | -2.6 | 16542 | 0.4 | 84995 | 2.8 |
| *miR-15b-5p* | 20482 | 3.82E-09 | 6766 | -1.5 | 1664 | -3.6 | 16374 | -0.2 | 157204 | 3.0 |
| *miR-16-5p* | 20495 | 5.64E-06 | 17938 | -0.1 | 9323 | -1.0 | 23256 | 0.3 | 118502 | 2.6 |
| *miR-181a-5p* | 3159 | 4.19E-04 | 3427 | 0.5 | 1017 | -1.2 | 2852 | 0.3 | 1809 | -0.4 |
| *miR-181c-5p* | 40 | 8.70E-05 | 47 | 0.3 | 2 | -4.7 | 57 | 0.7 | 40 | 0.1 |
| *miR-185-5p* | 592 | 4.15E-09 | 95 | -2.6 | 9 | -6.1 | 175 | -1.8 | 8544 | 3.9 |
| *miR-186-5p* | 189 | 4.80E-08 | 971 | 2.4 | 133 | -0.4 | 512 | 1.5 | 1936 | 3.4 |
| *miR-191-5p* | 8238 | 1.18E-06 | 1374 | -2.6 | 348 | -4.6 | 5705 | -0.5 | 5692 | -0.5 |
| *miR-194-5p* | 20 | 9.48E-10 | 181 | 3.2 | 5 | -2.5 | 85 | 2.1 | 3063 | 7.3 |
| *miR-197-3p* | 164 | 1.62E-04 | 41 | -2.1 | 4 | -6.1 | 572 | 1.6 | 33 | -2.2 |
| *miR-199a/b-3p** | 14497 | 1.21E-07 | 72 | -7.6 | 10 | -11.4 | 596 | -4.5 | 40 | -8.4 |
| *miR-19a-3p* | 214 | 1.69E-06 | 348 | 0.9 | 59 | -1.7 | 319 | 0.6 | 4950 | 4.7 |
| *miR-19b-3p* | 5496 | 2.24E-05 | 4395 | -0.1 | 1165 | -2.0 | 2446 | -1.0 | 10785 | 1.2 |
| *miR-200c-3p* | 52 | 5.42E-03 | 48 | -0.2 | 12 | -2.2 | 29 | -0.9 | 31 | -0.7 |
| *miR-206* | 21 | 2.26E-03 | 69 | 2.1 | 122 | 2.8 | 91 | 2.6 | 6 | -2.1 |
| *miR-20a/b-5p** | 8739 | 2.47E-05 | 7025 | -0.3 | 238 | -9.4 | 3792 | -1.3 | 27162 | 1.6 |
| *miR-21-5p* | 15814 | 1.20E-05 | 25829 | 0.8 | 2092 | -2.8 | 4237 | -1.7 | 12447 | -0.1 |
| *miR-222-3p* | 235 | 1.14E-08 | 1920 | 3.1 | 877 | 1.9 | 342 | 0.6 | 3495 | 3.9 |
| *miR-223-3p* | 83311 | 1.38E-06 | 8281 | -3.7 | 5249 | -4.1 | 449934 | 2.4 | 6075 | -3.7 |
| *miR-22-3p* | 1342 | 1.36E-07 | 300 | -2.2 | 12 | -7.4 | 367 | -1.8 | 6280 | 2.3 |
| *miR-23a-3p* | 13118 | 4.60E-07 | 1262 | -3.3 | 161 | -6.4 | 8890 | -0.5 | 164 | -6.2 |
| *miR-23b-3p* | 686 | 1.81E-06 | 67 | -3.4 | 5 | -7.3 | 237 | -1.6 | 125 | -2.5 |
| *miR-24-3p* | 8439 | 1.27E-08 | 1839 | -2.2 | 225 | -5.2 | 3291 | -1.3 | 269 | -5.0 |
| *miR-25-3p* | 7416 | 5.08E-08 | 5165 | -0.2 | 1683 | -1.8 | 10048 | 0.8 | 49547 | 3.1 |
| *miR-26a-5p* | 33364 | 1.36E-07 | 31141 | -0.1 | 4259 | -3.0 | 26292 | -0.3 | 3859 | -3.1 |
| *miR-26b-5p* | 604 | 5.39E-09 | 3315 | 2.5 | 682 | 0.2 | 1166 | 1.0 | 62081 | 6.8 |
| *miR-296-5p* | 83 | 1.68E-05 | 187 | 1.2 | 21 | -2.0 | 64 | -0.4 | 190 | 1.2 |
| *miR-29a-3p* | 2306 | 3.82E-07 | 13388 | 2.6 | 2738 | 0.2 | 9119 | 1.9 | 19 | -7.0 |
| *miR-29b-3p* | 93 | 9.90E-04 | 1461 | 4.2 | 150 | 0.5 | 516 | 2.7 | 353 | 2.2 |
| *miR-29c-3p* | 1583 | 1.36E-07 | 8568 | 2.5 | 2033 | 0.4 | 3783 | 1.3 | 1094 | -0.5 |
| *miR-301a-3p* | 356 | 5.90E-04 | 58 | -2.7 | 13 | -5.2 | 285 | -0.2 | 304 | -0.1 |
| *miR-30a-5p* | 404 | 7.26E-03 | 768 | 1.0 | 242 | -0.8 | 432 | 0.1 | 524 | 0.4 |
| *miR-30b-5p* | 4719 | 1.78E-07 | 7904 | 0.8 | 1765 | -1.4 | 2499 | -0.8 | 10143 | 1.2 |
| *miR-30c-5p* | 339 | 2.51E-04 | 356 | 0.1 | 57 | -2.6 | 685 | 1.1 | 751 | 1.2 |
| *miR-30d-5p* | 333 | 9.48E-10 | 534 | 0.7 | 126 | -1.4 | 340 | 0.1 | 3937 | 3.6 |
| *miR-30e-5p* | 147 | 6.44E-06 | 2453 | 4.1 | 584 | 1.9 | 1741 | 3.6 | 1924 | 3.8 |
| *miR-32-5p* | 107 | 2.91E-04 | 144 | 1.3 | 9 | -4.0 | 136 | 1.2 | 467 | 3.0 |
| *miR-331-3p* | 170 | 4.66E-05 | 68 | -1.3 | 18 | -3.8 | 56 | -1.6 | 361 | 1.1 |
| *miR-335-5p* | 1552 | 8.38E-07 | 131 | -3.7 | 22 | -6.8 | 125 | -3.6 | 49 | -4.9 |
| *miR-340-5p* | 1385 | 1.78E-07 | 314 | -2.3 | 38 | -5.2 | 2853 | 1.1 | 90 | -3.9 |
| *miR-342-3p* | 259 | 4.76E-09 | 21554 | 6.5 | 2614 | 3.4 | 506 | 1.0 | 428 | 0.8 |
| *miR-34c-5p* | 32 | 1.52E-06 | 33 | -0.7 | 4 | -3.7 | 40 | 0.3 | 412 | 3.7 |
| *miR-361-3p* | 57 | 5.90E-04 | 192 | 1.7 | 45 | -0.3 | 80 | 0.5 | 92 | 0.7 |
| *miR-361-5p* | 463 | 7.39E-07 | 235 | -1.0 | 22 | -4.5 | 439 | -0.1 | 40 | -3.5 |
| *miR-363-3p* | 72 | 3.98E-08 | 195 | 1.5 | 88 | 0.4 | 13 | -3.0 | 10674 | 7.3 |
| *miR-374a-5p* | 1609 | 1.11E-07 | 717 | -0.9 | 66 | -4.4 | 677 | -1.0 | 27360 | 4.4 |
| *miR-374b-5p* | 975 | 5.08E-08 | 478 | -1.0 | 43 | -4.5 | 609 | -0.7 | 4217 | 2.1 |
| *miR-423-3p* | 542 | 4.20E-05 | 334 | -0.7 | 52 | -3.4 | 291 | -0.8 | 116 | -2.3 |
| *miR-423-5p* | 2010 | 4.77E-06 | 596 | -1.7 | 234 | -3.0 | 834 | -1.2 | 2534 | 0.4 |
| *miR-425-5p* | 373 | 2.94E-07 | 179 | -1.0 | 51 | -2.9 | 601 | 0.7 | 2321 | 2.6 |
| *miR-451a* | 14217 | 4.15E-09 | 121 | -6.4 | 116 | -6.7 | 1067 | -3.6 | 1024619 | 6.7 |
| *miR-454-3p* | 97 | 3.82E-09 | 132 | 0.5 | 19 | -2.5 | 388 | 2.0 | 3788 | 5.3 |
| *miR-484* | 859 | 6.48E-08 | 234 | -1.9 | 40 | -4.4 | 427 | -1.2 | 665 | -0.4 |
| *miR-542-3p* | 44 | 3.09E-05 | 16 | -0.9 | 28 | -0.2 | 126 | 2.2 | 5 | -3.0 |
| *miR-548g-3p* | 70 | 1.49E-03 | 22 | -1.1 | 43 | 0.0 | 63 | -1.2 | 156 | 1.9 |
| *miR-660-5p* | 44 | 1.11E-07 | 69 | 0.7 | 30 | -0.6 | 51 | 0.2 | 1256 | 4.9 |
| *miR-720* | 3152 | 1.06E-06 | 11026 | 1.5 | 3147 | 0.0 | 7532 | 1.2 | 103 | -5.0 |
| *miR-92a-3p* | 19625 | 4.25E-06 | 14499 | -0.4 | 4998 | -1.9 | 4445 | -2.1 | 21592 | 0.2 |
| *miR-92b-3p* | 156 | 1.75E-05 | 89 | -0.8 | 24 | -2.9 | 47 | -1.9 | 2 | -6.7 |
| *miR-93-5p* | 853 | 1.65E-10 | 151 | -2.4 | 26 | -5.0 | 478 | -0.8 | 27493 | 5.1 |
| *miR-96-5p* | 664 | 4.66E-05 | 444 | -0.5 | 158 | -2.0 | 210 | -1.6 | 742 | 0.2 |
| *miR-98* | 1253 | 1.36E-07 | 199 | -2.6 | 22 | -6.0 | 232 | -2.3 | 1226 | 0.1 |
| *miR-99b-5p* | 303 | 1.70E-04 | 34 | -3.4 | 7 | -6.7 | 51 | -2.4 | 18 | -4.1 |

* In a few cases, the probes used were not able to distinguish between similar sequences miRNAs.

*q*-value shows the statistical significance, adjusted for multiple testing, for the differential expression of platelet miRNA compared to all other sell types.

**Table S6 B. miRNAs DE in T-cells compared with all other cell types.**

|  | Counts |  | Counts | Fold(Log2) | Counts | Fold(Log2) | Counts | Fold(Log2) | Counts | Fold(Log2) |
| --- | --- | --- | --- | --- | --- | --- | --- | --- | --- | --- |
| Gene Name | **T-cells** | ***q*-value** | **Platelets** | **Platelets *vs.* T-cells** | **B-cells** | **B-cells *vs*. T-cells** | **Granulo-**  **cytes** | **Gran. *vs*. T-cells** | **Erythro-**  **cytes** | **Erythr. *vs*. T-cells** |
| *let-7a-5p* | 8670 | 1.65E-10 | 18937 | 1.2 | 1796 | -2.2 | 4880 | -0.8 | 105131 | 3.7 |
| *let-7b-5p* | 1147 | 4.26E-10 | 1168 | 0.0 | 304 | -1.9 | 1020 | -0.2 | 56326 | 5.6 |
| *let-7c* | 132 | 1.01E-07 | 87 | -0.6 | 13 | -3.8 | 88 | -0.6 | 4830 | 5.3 |
| *let-7d-5p* | 3639 | 7.84E-05 | 15925 | 1.8 | 1157 | -1.7 | 3962 | 0.1 | 5032 | 0.5 |
| *let-7f-5p* | 5421 | 5.05E-07 | 13395 | 1.3 | 1133 | -2.2 | 2547 | -1.1 | 8348 | 0.7 |
| *let-7g-5p* | 55710 | 4.59E-07 | 21228 | -1.7 | 9844 | -2.5 | 9253 | -2.6 | 73217 | 0.4 |
| *let-7i-5p* | 3862 | 4.59E-07 | 11471 | 1.5 | 1421 | -1.5 | 1045 | -1.9 | 18282 | 2.2 |
| *miR-101-3p* | 2469 | 1.78E-07 | 567 | -2.4 | 458 | -2.6 | 1888 | -0.4 | 226 | -3.5 |
| *miR-103a-3p* | 2758 | 6.66E-09 | 12976 | 2.1 | 573 | -2.3 | 4774 | 0.8 | 142 | -4.3 |
| *miR-106a-5p/7-5p** | 252 | 2.56E-11 | 365 | 0.5 | 50 | -2.3 | 171 | -0.6 | 31312 | 7.0 |
| *miR-106b-5p* | 1870 | 5.56E-08 | 1607 | -0.3 | 538 | -1.8 | 3466 | 0.9 | 27992 | 3.9 |
| *miR-107* | 91 | 8.51E-07 | 891 | 3.6 | 12 | -3.1 | 263 | 1.9 | 2704 | 5.3 |
| *miR-10a-5p* | 76 | 2.61E-02 | 109 | 0.3 | 22 | -2.4 | 42 | -1.6 | 11 | -4.0 |
| *miR-125b-5p* | 463 | 1.14E-03 | 162 | -1.3 | 9 | -6.6 | 53 | -2.9 | 182 | -1.3 |
| *miR-1260a* | 3816 | 3.05E-06 | 185 | -4.4 | 2105 | -0.8 | 1222 | -1.8 | 127 | -5.0 |
| *miR-126-3p* | 450 | 3.37E-08 | 85658 | 7.4 | 85 | -2.5 | 148 | -1.6 | 32963 | 6.2 |
| *miR-128* | 49 | 2.73E-04 | 151 | 1.6 | 15 | -2.5 | 118 | 1.2 | 123 | 1.3 |
| *miR-1283* | 72 | 7.84E-05 | 6 | -3.7 | 124 | 0.7 | 87 | 0.3 | 238 | 1.8 |
| *miR-130b-3p* | 26 | 2.36E-04 | 153 | 2.6 | 4 | -3.4 | 39 | 0.7 | 63 | 1.4 |
| *miR-140-3p* | 366 | 6.86E-07 | 21 | -4.2 | 91 | -2.1 | 72 | -2.4 | 142 | -1.4 |
| *miR-140-5p* | 429 | 8.38E-07 | 253 | -0.8 | 61 | -2.8 | 860 | 1.0 | 475 | 0.1 |
| *miR-142-3p* | 119999 | 2.73E-04 | 67408 | -1.0 | 30119 | -2.2 | 112362 | -0.1 | 35909 | -1.7 |
| *miR-142-5p* | 20928 | 2.12E-07 | 2167 | -3.3 | 3175 | -3.1 | 18903 | -0.1 | 37 | -9.3 |
| *miR-146a-5p* | 3614 | 3.50E-09 | 3964 | 0.2 | 325 | -3.6 | 31 | -6.9 | 76 | -5.6 |
| *miR-146b-5p* | 2186 | 4.56E-07 | 40 | -5.8 | 34 | -7.7 | 18 | -6.9 | 96 | -4.5 |
| *miR-148a-3p* | 426 | 2.01E-07 | 1446 | 1.7 | 101 | -2.2 | 4168 | 3.3 | 2035 | 2.2 |
| *miR-148b-3p* | 507 | 9.52E-07 | 3993 | 2.9 | 138 | -2.0 | 1531 | 1.6 | 1597 | 1.7 |
| *miR-150-5p* | 207293 | 2.56E-11 | 38 | -12.5 | 28880 | -2.9 | 1090 | -7.7 | 90 | -11.2 |
| *miR-1537* | 122 | 4.59E-07 | 99 | -0.5 | 27 | -1.9 | 1687 | 4.1 | 47 | -1.1 |
| *miR-155-5p* | 711 | 3.55E-07 | 37 | -4.3 | 237 | -1.7 | 17 | -5.7 | 97 | -2.9 |
| *miR-15a-5p* | 4075 | 1.78E-07 | 14117 | 1.6 | 2009 | -1.0 | 16542 | 2.0 | 84995 | 4.4 |
| *miR-15b-5p* | 6766 | 3.82E-09 | 20482 | 1.5 | 1664 | -2.0 | 16374 | 1.3 | 157204 | 4.5 |
| *miR-16-5p* | 17938 | 5.64E-06 | 20495 | 0.1 | 9323 | -1.0 | 23256 | 0.4 | 118502 | 2.7 |
| *miR-181a-5p* | 3427 | 4.19E-04 | 3159 | -0.5 | 1017 | -1.7 | 2852 | -0.3 | 1809 | -0.9 |
| *miR-181c-5p* | 47 | 8.70E-05 | 40 | -0.3 | 2 | -4.9 | 57 | 0.4 | 40 | -0.2 |
| *miR-185-5p* | 95 | 4.15E-09 | 592 | 2.6 | 9 | -3.5 | 175 | 0.9 | 8544 | 6.5 |
| *miR-186-5p* | 971 | 4.80E-08 | 189 | -2.4 | 133 | -2.9 | 512 | -0.9 | 1936 | 1.0 |
| *miR-191-5p* | 1374 | 1.18E-06 | 8238 | 2.6 | 348 | -2.0 | 5705 | 2.1 | 5692 | 2.1 |
| *miR-194-5p* | 181 | 9.48E-10 | 20 | -3.2 | 5 | -5.7 | 85 | -1.1 | 3063 | 4.1 |
| *miR-197-3p* | 41 | 1.62E-04 | 164 | 2.1 | 4 | -4.0 | 572 | 3.6 | 33 | -0.1 |
| *miR-199a/b-3p** | 72 | 1.21E-07 | 14497 | 7.6 | 10 | -3.8 | 596 | 3.0 | 40 | -0.8 |
| *miR-19a-3p* | 348 | 1.69E-06 | 214 | -0.9 | 59 | -2.6 | 319 | -0.3 | 4950 | 3.8 |
| *miR-19b-3p* | 4395 | 2.24E-05 | 5496 | 0.1 | 1165 | -1.9 | 2446 | -0.9 | 10785 | 1.3 |
| *miR-200c-3p* | 48 | 5.42E-03 | 52 | 0.2 | 12 | -2.0 | 29 | -0.7 | 31 | -0.6 |
| *miR-206* | 69 | 2.26E-03 | 21 | -2.1 | 122 | 0.7 | 91 | 0.5 | 6 | -4.2 |
| *miR-20a/b-5p** | 7025 | 2.47E-05 | 8739 | 0.3 | 238 | -9.1 | 3792 | -0.9 | 27162 | 2.0 |
| *miR-21-5p* | 25829 | 1.20E-05 | 15814 | -0.8 | 2092 | -3.6 | 4237 | -2.6 | 12447 | -1.0 |
| *miR-222-3p* | 1920 | 1.14E-08 | 235 | -3.1 | 877 | -1.2 | 342 | -2.5 | 3495 | 0.9 |
| *miR-223-3p* | 8281 | 1.38E-06 | 83311 | 3.7 | 5249 | -0.3 | 449934 | 6.2 | 6075 | 0.1 |
| *miR-22-3p* | 300 | 1.36E-07 | 1342 | 2.2 | 12 | -5.3 | 367 | 0.3 | 6280 | 4.4 |
| *miR-23a-3p* | 1262 | 4.60E-07 | 13118 | 3.3 | 161 | -3.1 | 8890 | 2.8 | 164 | -3.0 |
| *miR-23b-3p* | 67 | 1.81E-06 | 686 | 3.4 | 5 | -3.9 | 237 | 1.8 | 125 | 0.9 |
| *miR-24-3p* | 1839 | 1.27E-08 | 8439 | 2.2 | 225 | -3.0 | 3291 | 0.9 | 269 | -2.8 |
| *miR-25-3p* | 5165 | 5.08E-08 | 7416 | 0.2 | 1683 | -1.6 | 10048 | 1.0 | 49547 | 3.3 |
| *miR-26a-5p* | 31141 | 1.36E-07 | 33364 | 0.1 | 4259 | -2.8 | 26292 | -0.2 | 3859 | -3.0 |
| *miR-26b-5p* | 3315 | 5.39E-09 | 604 | -2.5 | 682 | -2.3 | 1166 | -1.5 | 62081 | 4.3 |
| *miR-296-5p* | 187 | 1.68E-05 | 83 | -1.2 | 21 | -3.1 | 64 | -1.5 | 190 | 0.0 |
| *miR-29a-3p* | 13388 | 3.82E-07 | 2306 | -2.6 | 2738 | -2.4 | 9119 | -0.7 | 19 | -9.6 |
| *miR-29b-3p* | 1461 | 9.90E-04 | 93 | -4.2 | 150 | -3.7 | 516 | -1.5 | 353 | -1.9 |
| *miR-29c-3p* | 8568 | 1.36E-07 | 1583 | -2.5 | 2033 | -2.1 | 3783 | -1.2 | 1094 | -3.0 |
| *miR-301a-3p* | 58 | 5.90E-04 | 356 | 2.7 | 13 | -2.5 | 285 | 2.5 | 304 | 2.5 |
| *miR-30a-5p* | 768 | 7.26E-03 | 404 | -1.0 | 242 | -1.8 | 432 | -0.9 | 524 | -0.5 |
| *miR-30b-5p* | 7904 | 1.78E-07 | 4719 | -0.8 | 1765 | -2.2 | 2499 | -1.6 | 10143 | 0.4 |
| *miR-30c-5p* | 356 | 2.51E-04 | 339 | -0.1 | 57 | -2.7 | 685 | 1.0 | 751 | 1.2 |
| *miR-30d-5p* | 534 | 9.48E-10 | 333 | -0.7 | 126 | -2.0 | 340 | -0.6 | 3937 | 2.9 |
| *miR-30e-5p* | 2453 | 6.44E-06 | 147 | -4.1 | 584 | -2.2 | 1741 | -0.5 | 1924 | -0.4 |
| *miR-32-5p* | 144 | 2.91E-04 | 107 | -1.3 | 9 | -5.2 | 136 | -0.1 | 467 | 1.8 |
| *miR-331-3p* | 68 | 4.66E-05 | 170 | 1.3 | 18 | -2.5 | 56 | -0.3 | 361 | 2.5 |
| *miR-335-5p* | 131 | 8.38E-07 | 1552 | 3.7 | 22 | -3.1 | 125 | 0.1 | 49 | -1.2 |
| *miR-340-5p* | 314 | 1.78E-07 | 1385 | 2.3 | 38 | -2.9 | 2853 | 3.4 | 90 | -1.6 |
| *miR-342-3p* | 21554 | 4.76E-09 | 259 | -6.5 | 2614 | -3.1 | 506 | -5.5 | 428 | -5.6 |
| *miR-34c-5p* | 33 | 1.52E-06 | 32 | 0.7 | 4 | -3.0 | 40 | 1.0 | 412 | 4.4 |
| *miR-361-3p* | 192 | 5.90E-04 | 57 | -1.7 | 45 | -2.0 | 80 | -1.2 | 92 | -1.0 |
| *miR-361-5p* | 235 | 7.39E-07 | 463 | 1.0 | 22 | -3.5 | 439 | 0.9 | 40 | -2.5 |
| *miR-363-3p* | 195 | 3.98E-08 | 72 | -1.5 | 88 | -1.1 | 13 | -4.5 | 10674 | 5.8 |
| *miR-374a-5p* | 717 | 1.11E-07 | 1609 | 0.9 | 66 | -3.5 | 677 | -0.1 | 27360 | 5.3 |
| *miR-374b-5p* | 478 | 5.08E-08 | 975 | 1.0 | 43 | -3.5 | 609 | 0.4 | 4217 | 3.2 |
| *miR-423-3p* | 334 | 4.20E-05 | 542 | 0.7 | 52 | -2.7 | 291 | -0.2 | 116 | -1.6 |
| *miR-423-5p* | 596 | 4.77E-06 | 2010 | 1.7 | 234 | -1.3 | 834 | 0.5 | 2534 | 2.1 |
| *miR-425-5p* | 179 | 2.94E-07 | 373 | 1.0 | 51 | -1.9 | 601 | 1.7 | 2321 | 3.7 |
| *miR-451a* | 121 | 4.15E-09 | 14217 | 6.4 | 116 | -0.3 | 1067 | 2.9 | 1024619 | 13.1 |
| *miR-454-3p* | 132 | 3.82E-09 | 97 | -0.5 | 19 | -3.0 | 388 | 1.5 | 3788 | 4.8 |
| *miR-484* | 234 | 6.48E-08 | 859 | 1.9 | 40 | -2.6 | 427 | 0.6 | 665 | 1.5 |
| *miR-542-3p* | 16 | 3.09E-05 | 44 | 0.9 | 28 | 0.7 | 126 | 3.1 | 5 | -2.1 |
| *miR-548g-3p* | 22 | 1.49E-03 | 70 | 1.1 | 43 | 1.1 | 63 | -0.1 | 156 | 3.0 |
| *miR-660-5p* | 69 | 1.11E-07 | 44 | -0.7 | 30 | -1.3 | 51 | -0.5 | 1256 | 4.2 |
| *miR-720* | 11026 | 1.06E-06 | 3152 | -1.5 | 3147 | -1.5 | 7532 | -0.3 | 103 | -6.5 |
| *miR-92a-3p* | 14499 | 4.25E-06 | 19625 | 0.4 | 4998 | -1.5 | 4445 | -1.7 | 21592 | 0.6 |
| *miR-92b-3p* | 89 | 1.75E-05 | 156 | 0.8 | 24 | -2.1 | 47 | -1.0 | 2 | -5.9 |
| *miR-93-5p* | 151 | 1.65E-10 | 853 | 2.4 | 26 | -2.6 | 478 | 1.6 | 27493 | 7.5 |
| *miR-96-5p* | 444 | 4.66E-05 | 664 | 0.5 | 158 | -1.5 | 210 | -1.1 | 742 | 0.7 |
| *miR-98* | 199 | 1.36E-07 | 1253 | 2.6 | 22 | -3.4 | 232 | 0.2 | 1226 | 2.6 |
| *miR-99b-5p* | 34 | 1.70E-04 | 303 | 3.4 | 7 | -3.3 | 51 | 0.9 | 18 | -0.7 |

* In a few cases, the probes used were not able to distinguish between similar sequences miRNAs.

*q*-value shows the statistical significance, adjusted for multiple testing, for the differential expression of T-cells miRNA compared to all other sell types.

**Table S6 C. miRNAs DE in B-cells compared with all other cell types.**

|  | Counts |  | Counts | Fold(Log2) | Counts | Fold(Log2) | Counts | Fold(Log2) |  | Fold(Log2) |
| --- | --- | --- | --- | --- | --- | --- | --- | --- | --- | --- |
| Gene Name | **B-cells** | ***q*-value** | **Platelets** | **Platelets *vs.* B-cells** | **T-cells** | **T-cells *vs*. B-cells** | **Granulo-**  **cytes** | **Gran. *vs*. B-cells** | **Erythro-**  **cytes** | **Erythr. *vs.* B-cells** |
| *let-7a-5p* | 1796 | 1.65E-10 | 18937 | 3.4 | 8670 | 2.2 | 4880 | 1.4 | 105131 | 5.9 |
| *let-7b-5p* | 304 | 4.26E-10 | 1168 | 1.9 | 1147 | 1.9 | 1020 | 1.8 | 56326 | 7.6 |
| *let-7c* | 13 | 1.01E-07 | 87 | 3.2 | 132 | 3.8 | 88 | 3.2 | 4830 | 9.1 |
| *let-7d-5p* | 1157 | 7.84E-05 | 15925 | 3.5 | 3639 | 1.7 | 3962 | 1.8 | 5032 | 2.1 |
| *let-7f-5p* | 1133 | 5.05E-07 | 13395 | 3.5 | 5421 | 2.2 | 2547 | 1.1 | 8348 | 2.9 |
| *let-7g-5p* | 9844 | 4.59E-07 | 21228 | 0.9 | 55710 | 2.5 | 9253 | -0.1 | 73217 | 2.9 |
| *let-7i-5p* | 1421 | 4.59E-07 | 11471 | 3.0 | 3862 | 1.5 | 1045 | -0.4 | 18282 | 3.7 |
| *miR-101-3p* | 458 | 1.78E-07 | 567 | 0.2 | 2469 | 2.6 | 1888 | 2.2 | 226 | -0.9 |
| *miR-103a-3p* | 573 | 6.66E-09 | 12976 | 4.4 | 2758 | 2.3 | 4774 | 3.1 | 142 | -2.1 |
| *miR-106a-5p/17-5p** | 50 | 2.56E-11 | 365 | 2.9 | 252 | 2.3 | 171 | 1.8 | 31312 | 9.3 |
| *miR-106b-5p* | 538 | 5.56E-08 | 1607 | 1.6 | 1870 | 1.8 | 3466 | 2.7 | 27992 | 5.8 |
| *miR-107* | 12 | 8.51E-07 | 891 | 6.8 | 91 | 3.1 | 263 | 5.1 | 2704 | 8.4 |
| *miR-10a-5p* | 22 | 2.61E-02 | 109 | 2.6 | 76 | 2.4 | 42 | 0.8 | 11 | -1.6 |
| *miR-125b-5p* | 9 | 1.14E-03 | 162 | 5.3 | 463 | 6.6 | 53 | 3.7 | 182 | 5.3 |
| *miR-1260a* | 2105 | 3.05E-06 | 185 | -3.6 | 3816 | 0.8 | 1222 | -1.0 | 127 | -4.2 |
| *miR-126-3p* | 85 | 3.37E-08 | 85658 | 9.9 | 450 | 2.5 | 148 | 0.9 | 32963 | 8.7 |
| *miR-128* | 15 | 2.73E-04 | 151 | 4.2 | 49 | 2.5 | 118 | 3.8 | 123 | 3.9 |
| *miR-1283* | 124 | 7.84E-05 | 6 | -4.3 | 72 | -0.7 | 87 | -0.4 | 238 | 1.1 |
| *miR-130b-3p* | 4 | 2.36E-04 | 153 | 6.0 | 26 | 3.4 | 39 | 4.0 | 63 | 4.7 |
| *miR-140-3p* | 91 | 6.86E-07 | 21 | -2.2 | 366 | 2.1 | 72 | -0.3 | 142 | 0.7 |
| *miR-140-5p* | 61 | 8.38E-07 | 253 | 2.0 | 429 | 2.8 | 860 | 3.8 | 475 | 3.0 |
| *miR-142-3p* | 30119 | 2.73E-04 | 67408 | 1.2 | 119999 | 2.2 | 112362 | 2.1 | 35909 | 0.5 |
| *miR-142-5p* | 3175 | 2.12E-07 | 2167 | -0.2 | 20928 | 3.1 | 18903 | 3.0 | 37 | -6.2 |
| *miR-146a-5p* | 325 | 3.50E-09 | 3964 | 3.8 | 3614 | 3.6 | 31 | -3.3 | 76 | -2.0 |
| *miR-146b-5p* | 34 | 4.56E-07 | 40 | 1.9 | 2186 | 7.7 | 18 | 0.8 | 96 | 3.2 |
| *miR-148a-3p* | 101 | 2.01E-07 | 1446 | 4.0 | 426 | 2.2 | 4168 | 5.5 | 2035 | 4.5 |
| *miR-148b-3p* | 138 | 9.52E-07 | 3993 | 4.9 | 507 | 2.0 | 1531 | 3.6 | 1597 | 3.6 |
| *miR-150-5p* | 28880 | 2.56E-11 | 38 | -9.7 | 207293 | 2.9 | 1090 | -4.8 | 90 | -8.3 |
| *miR-1537* | 27 | 4.59E-07 | 99 | 1.3 | 122 | 1.9 | 1687 | 6.0 | 47 | 0.8 |
| *miR-155-5p* | 237 | 3.55E-07 | 37 | -2.6 | 711 | 1.7 | 17 | -4.0 | 97 | -1.2 |
| *miR-15a-5p* | 2009 | 1.78E-07 | 14117 | 2.6 | 4075 | 1.0 | 16542 | 3.0 | 84995 | 5.4 |
| *miR-15b-5p* | 1664 | 3.82E-09 | 20482 | 3.6 | 6766 | 2.0 | 16374 | 3.3 | 157204 | 6.6 |
| *miR-16-5p* | 9323 | 5.64E-06 | 20495 | 1.0 | 17938 | 1.0 | 23256 | 1.3 | 118502 | 3.6 |
| *miR-181a-5p* | 1017 | 4.19E-04 | 3159 | 1.2 | 3427 | 1.7 | 2852 | 1.4 | 1809 | 0.8 |
| *miR-181c-5p* | 2 | 8.70E-05 | 40 | 4.7 | 47 | 4.9 | 57 | 5.3 | 40 | 4.7 |
| *miR-185-5p* | 9 | 4.15E-09 | 592 | 6.1 | 95 | 3.5 | 175 | 4.3 | 8544 | 10.0 |
| *miR-186-5p* | 133 | 4.80E-08 | 189 | 0.4 | 971 | 2.9 | 512 | 1.9 | 1936 | 3.9 |
| *miR-191-5p* | 348 | 1.18E-06 | 8238 | 4.6 | 1374 | 2.0 | 5705 | 4.1 | 5692 | 4.1 |
| *miR-194-5p* | 5 | 9.48E-10 | 20 | 2.5 | 181 | 5.7 | 85 | 4.6 | 3063 | 9.8 |
| *miR-197-3p* | 4 | 1.62E-04 | 164 | 6.1 | 41 | 4.0 | 572 | 7.7 | 33 | 3.9 |
| *miR-199a/a-3p** | 10 | 1.21E-07 | 14497 | 11.4 | 72 | 3.8 | 596 | 6.9 | 40 | 3.0 |
| *miR-19a-3p* | 59 | 1.69E-06 | 214 | 1.7 | 348 | 2.6 | 319 | 2.3 | 4950 | 6.4 |
| *miR-19b-3p* | 1165 | 2.24E-05 | 5496 | 2.0 | 4395 | 1.9 | 2446 | 1.1 | 10785 | 3.2 |
| *miR-200c-3p* | 12 | 5.42E-03 | 52 | 2.2 | 48 | 2.0 | 29 | 1.3 | 31 | 1.5 |
| *miR-206* | 122 | 2.26E-03 | 21 | -2.8 | 69 | -0.7 | 91 | -0.2 | 6 | -4.9 |
| *miR-20a/b-5p** | 238 | 2.47E-05 | 8739 | 9.4 | 7025 | 9.1 | 3792 | 8.2 | 27162 | 11.0 |
| *miR-21-5p* | 2092 | 1.20E-05 | 15814 | 2.8 | 25829 | 3.6 | 4237 | 1.1 | 12447 | 2.6 |
| *miR-222-3p* | 877 | 1.14E-08 | 235 | -1.9 | 1920 | 1.2 | 342 | -1.3 | 3495 | 2.1 |
| *miR-223-3p* | 5249 | 1.38E-06 | 83311 | 4.1 | 8281 | 0.3 | 449934 | 6.5 | 6075 | 0.4 |
| *miR-22-3p* | 12 | 1.36E-07 | 1342 | 7.4 | 300 | 5.3 | 367 | 5.6 | 6280 | 9.7 |
| *miR-23a-3p* | 161 | 4.60E-07 | 13118 | 6.4 | 1262 | 3.1 | 8890 | 5.9 | 164 | 0.1 |
| *miR-23b-3p* | 5 | 1.81E-06 | 686 | 7.3 | 67 | 3.9 | 237 | 5.7 | 125 | 4.8 |
| *miR-24-3p* | 225 | 1.27E-08 | 8439 | 5.2 | 1839 | 3.0 | 3291 | 3.9 | 269 | 0.2 |
| *miR-25-3p* | 1683 | 5.08E-08 | 7416 | 1.8 | 5165 | 1.6 | 10048 | 2.6 | 49547 | 4.9 |
| *miR-26a-5p* | 4259 | 1.36E-07 | 33364 | 3.0 | 31141 | 2.8 | 26292 | 2.6 | 3859 | -0.1 |
| *miR-26b-5p* | 682 | 5.39E-09 | 604 | -0.2 | 3315 | 2.3 | 1166 | 0.8 | 62081 | 6.6 |
| *miR-296-5p* | 21 | 1.68E-05 | 83 | 2.0 | 187 | 3.1 | 64 | 1.6 | 190 | 3.2 |
| *miR-29a-3p* | 2738 | 3.82E-07 | 2306 | -0.2 | 13388 | 2.4 | 9119 | 1.7 | 19 | -7.2 |
| *miR-29b-3p* | 150 | 9.90E-04 | 93 | -0.5 | 1461 | 3.7 | 516 | 2.2 | 353 | 1.7 |
| *miR-29c-3p* | 2033 | 1.36E-07 | 1583 | -0.4 | 8568 | 2.1 | 3783 | 1.0 | 1094 | -0.9 |
| *miR-301a-3p* | 13 | 5.90E-04 | 356 | 5.2 | 58 | 2.5 | 285 | 5.0 | 304 | 5.1 |
| *miR-30a-5p* | 242 | 7.26E-03 | 404 | 0.8 | 768 | 1.8 | 432 | 0.9 | 524 | 1.2 |
| *miR-30b-5p* | 1765 | 1.78E-07 | 4719 | 1.4 | 7904 | 2.2 | 2499 | 0.5 | 10143 | 2.5 |
| *miR-30c-5p* | 57 | 2.51E-04 | 339 | 2.6 | 356 | 2.7 | 685 | 3.7 | 751 | 3.9 |
| *miR-30d-5p* | 126 | 9.48E-10 | 333 | 1.4 | 534 | 2.0 | 340 | 1.4 | 3937 | 5.0 |
| *miR-30e-5p* | 584 | 6.44E-06 | 147 | -1.9 | 2453 | 2.2 | 1741 | 1.7 | 1924 | 1.9 |
| *miR-32-5p* | 9 | 2.91E-04 | 107 | 4.0 | 144 | 5.2 | 136 | 5.2 | 467 | 7.0 |
| *miR-331-3p* | 18 | 4.66E-05 | 170 | 3.8 | 68 | 2.5 | 56 | 2.2 | 361 | 5.0 |
| *miR-335-5p* | 22 | 8.38E-07 | 1552 | 6.8 | 131 | 3.1 | 125 | 3.2 | 49 | 1.8 |
| *miR-340-5p* | 38 | 1.78E-07 | 1385 | 5.2 | 314 | 2.9 | 2853 | 6.3 | 90 | 1.3 |
| *miR-342-3p* | 2614 | 4.76E-09 | 259 | -3.4 | 21554 | 3.1 | 506 | -2.4 | 428 | -2.6 |
| *miR-34c-5p* | 4 | 1.52E-06 | 32 | 3.7 | 33 | 3.0 | 40 | 4.0 | 412 | 7.4 |
| *miR-361-3p* | 45 | 5.90E-04 | 57 | 0.3 | 192 | 2.0 | 80 | 0.8 | 92 | 1.0 |
| *miR-361-5p* | 22 | 7.39E-07 | 463 | 4.5 | 235 | 3.5 | 439 | 4.4 | 40 | 1.0 |
| *miR-363-3p* | 88 | 3.98E-08 | 72 | -0.4 | 195 | 1.1 | 13 | -3.3 | 10674 | 7.0 |
| *miR-374a-5p* | 66 | 1.11E-07 | 1609 | 4.4 | 717 | 3.5 | 677 | 3.5 | 27360 | 8.8 |
| *miR-374b-5p* | 43 | 5.08E-08 | 975 | 4.5 | 478 | 3.5 | 609 | 3.9 | 4217 | 6.7 |
| *miR-423-3p* | 52 | 4.20E-05 | 542 | 3.4 | 334 | 2.7 | 291 | 2.5 | 116 | 1.1 |
| *miR-423-5p* | 234 | 4.77E-06 | 2010 | 3.0 | 596 | 1.3 | 834 | 1.9 | 2534 | 3.5 |
| *miR-425-5p* | 51 | 2.94E-07 | 373 | 2.9 | 179 | 1.9 | 601 | 3.6 | 2321 | 5.5 |
| *miR-451a* | 116 | 4.15E-09 | 14217 | 6.7 | 121 | 0.3 | 1067 | 3.1 | 1024619 | 13.4 |
| *miR-454-3p* | 19 | 3.82E-09 | 97 | 2.5 | 132 | 3.0 | 388 | 4.5 | 3788 | 7.8 |
| *miR-484* | 40 | 6.48E-08 | 859 | 4.4 | 234 | 2.6 | 427 | 3.2 | 665 | 4.1 |
| *miR-542-3p* | 28 | 3.09E-05 | 44 | 0.2 | 16 | -0.7 | 126 | 2.4 | 5 | -2.8 |
| *miR-548g-3p* | 43 | 1.49E-03 | 70 | 0.0 | 22 | -1.1 | 63 | -1.2 | 156 | 1.9 |
| *miR-660-5p* | 30 | 1.11E-07 | 44 | 0.6 | 69 | 1.3 | 51 | 0.8 | 1256 | 5.5 |
| *miR-720* | 3147 | 1.06E-06 | 3152 | 0.0 | 11026 | 1.5 | 7532 | 1.3 | 103 | -5.0 |
| *miR-92a-3p* | 4998 | 4.25E-06 | 19625 | 1.9 | 14499 | 1.5 | 4445 | -0.2 | 21592 | 2.1 |
| *miR-92b-3p* | 24 | 1.75E-05 | 156 | 2.9 | 89 | 2.1 | 47 | 1.0 | 2 | -3.8 |
| *miR-93-5p* | 26 | 1.65E-10 | 853 | 5.0 | 151 | 2.6 | 478 | 4.3 | 27493 | 10.1 |
| *miR-96-5p* | 158 | 4.66E-05 | 664 | 2.0 | 444 | 1.5 | 210 | 0.4 | 742 | 2.3 |
| *miR-98* | 22 | 1.36E-07 | 1253 | 6.0 | 199 | 3.4 | 232 | 3.7 | 1226 | 6.1 |
| *miR-99b-5p* | 7 | 1.70E-04 | 303 | 6.7 | 34 | 3.3 | 51 | 4.3 | 18 | 2.6 |

* In a few cases, the probes used were not able to distinguish between similar sequences miRNAs.

*q*-value shows the statistical significance, adjusted for multiple testing, for the differential expression of B-cells miRNA compared to all other sell types.

**Table S6 D. miRNAs DE in granulocytes compared with all other cell types.**

|  | Counts |  | Counts | Fold(Log2) | Counts | Fold(Log2) | Counts | Fold(Log2) | Counts | Fold(Log2) |
| --- | --- | --- | --- | --- | --- | --- | --- | --- | --- | --- |
| Gene Name | **Granulo-**  **cytes** | **q-value** | **Platelets** | **Platelets vs. Gran.** | **T-cells** | **T-cells vs. Gran.** | **B-cells** | **B-cells vs. Gran.** | **Erythro-**  **cytes** | **Eythr. vs. Gran.** |
| let-7a-5p | 4880 | 1.65E-10 | 18937 | 1.9 | 8670 | 0.8 | 1796 | -1.4 | 105131 | 4.4 |
| let-7b-5p | 1020 | 4.26E-10 | 1168 | 0.2 | 1147 | 0.2 | 304 | -1.8 | 56326 | 5.8 |
| let-7c | 88 | 1.01E-07 | 87 | 0.0 | 132 | 0.6 | 13 | -3.2 | 4830 | 5.9 |
| let-7d-5p | 3962 | 7.84E-05 | 15925 | 1.7 | 3639 | -0.1 | 1157 | -1.8 | 5032 | 0.3 |
| let-7f-5p | 2547 | 5.05E-07 | 13395 | 2.4 | 5421 | 1.1 | 1133 | -1.1 | 8348 | 1.8 |
| let-7g-5p | 9253 | 4.59E-07 | 21228 | 0.9 | 55710 | 2.6 | 9844 | 0.1 | 73217 | 3.0 |
| let-7i-5p | 1045 | 4.59E-07 | 11471 | 3.4 | 3862 | 1.9 | 1421 | 0.4 | 18282 | 4.1 |
| miR-101-3p | 1888 | 1.78E-07 | 567 | -2.0 | 2469 | 0.4 | 458 | -2.2 | 226 | -3.0 |
| miR-103a-3p | 4774 | 6.66E-09 | 12976 | 1.3 | 2758 | -0.8 | 573 | -3.1 | 142 | -5.1 |
| miR-106a-5p/17-5p* | 171 | 2.56E-11 | 365 | 1.1 | 252 | 0.6 | 50 | -1.8 | 31312 | 7.5 |
| miR-106b-5p | 3466 | 5.56E-08 | 1607 | -1.1 | 1870 | -0.9 | 538 | -2.7 | 27992 | 3.0 |
| miR-107 | 263 | 8.51E-07 | 891 | 1.7 | 91 | -1.9 | 12 | -5.1 | 2704 | 3.4 |
| miR-10a-5p | 42 | 2.61E-02 | 109 | 1.8 | 76 | 1.6 | 22 | -0.8 | 11 | -2.4 |
| miR-125b-5p | 53 | 1.14E-03 | 162 | 1.6 | 463 | 2.9 | 9 | -3.7 | 182 | 1.6 |
| miR-1260a | 1222 | 3.05E-06 | 185 | -2.6 | 3816 | 1.8 | 2105 | 1.0 | 127 | -3.2 |
| miR-126-3p | 148 | 3.37E-08 | 85658 | 9.0 | 450 | 1.6 | 85 | -0.9 | 32963 | 7.9 |
| miR-128 | 118 | 2.73E-04 | 151 | 0.4 | 49 | -1.2 | 15 | -3.8 | 123 | 0.1 |
| miR-1283 | 87 | 7.84E-05 | 6 | -4.0 | 72 | -0.3 | 124 | 0.4 | 238 | 1.5 |
| miR-130b-3p | 39 | 2.36E-04 | 153 | 2.0 | 26 | -0.7 | 4 | -4.0 | 63 | 0.7 |
| miR-140-3p | 72 | 6.86E-07 | 21 | -1.9 | 366 | 2.4 | 91 | 0.3 | 142 | 1.0 |
| miR-140-5p | 860 | 8.38E-07 | 253 | -1.8 | 429 | -1.0 | 61 | -3.8 | 475 | -0.8 |
| miR-142-3p | 112362 | 2.73E-04 | 67408 | -0.9 | 119999 | 0.1 | 30119 | -2.1 | 35909 | -1.7 |
| miR-142-5p | 18903 | 2.12E-07 | 2167 | -3.2 | 20928 | 0.1 | 3175 | -3.0 | 37 | -9.2 |
| miR-146a-5p | 31 | 3.50E-09 | 3964 | 7.0 | 3614 | 6.9 | 325 | 3.3 | 76 | 1.3 |
| miR-146b-5p | 18 | 4.56E-07 | 40 | 1.1 | 2186 | 6.9 | 34 | -0.8 | 96 | 2.4 |
| miR-148a-3p | 4168 | 2.01E-07 | 1446 | -1.6 | 426 | -3.3 | 101 | -5.5 | 2035 | -1.0 |
| miR-148b-3p | 1531 | 9.52E-07 | 3993 | 1.3 | 507 | -1.6 | 138 | -3.6 | 1597 | 0.1 |
| miR-150-5p | 1090 | 2.56E-11 | 38 | -4.9 | 207293 | 7.7 | 28880 | 4.8 | 90 | -3.5 |
| miR-1537 | 1687 | 4.59E-07 | 99 | -4.7 | 122 | -4.1 | 27 | -6.0 | 47 | -5.2 |
| miR-155-5p | 17 | 3.55E-07 | 37 | 1.4 | 711 | 5.7 | 237 | 4.0 | 97 | 2.8 |
| miR-15a-5p | 16542 | 1.78E-07 | 14117 | -0.4 | 4075 | -2.0 | 2009 | -3.0 | 84995 | 2.4 |
| miR-15b-5p | 16374 | 3.82E-09 | 20482 | 0.2 | 6766 | -1.3 | 1664 | -3.3 | 157204 | 3.3 |
| miR-16-5p | 23256 | 5.64E-06 | 20495 | -0.3 | 17938 | -0.4 | 9323 | -1.3 | 118502 | 2.3 |
| miR-181a-5p | 2852 | 4.19E-04 | 3159 | -0.3 | 3427 | 0.3 | 1017 | -1.4 | 1809 | -0.7 |
| miR-181c-5p | 57 | 8.70E-05 | 40 | -0.7 | 47 | -0.4 | 2 | -5.3 | 40 | -0.6 |
| miR-185-5p | 175 | 4.15E-09 | 592 | 1.8 | 95 | -0.9 | 9 | -4.3 | 8544 | 5.6 |
| miR-186-5p | 512 | 4.80E-08 | 189 | -1.5 | 971 | 0.9 | 133 | -1.9 | 1936 | 1.9 |
| miR-191-5p | 5705 | 1.18E-06 | 8238 | 0.5 | 1374 | -2.1 | 348 | -4.1 | 5692 | 0.0 |
| miR-194-5p | 85 | 9.48E-10 | 20 | -2.1 | 181 | 1.1 | 5 | -4.6 | 3063 | 5.2 |
| miR-197-3p | 572 | 1.62E-04 | 164 | -1.6 | 41 | -3.6 | 4 | -7.7 | 33 | -3.8 |
| miR-199a/b-3p* | 596 | 1.21E-07 | 14497 | 4.5 | 72 | -3.0 | 10 | -6.9 | 40 | -3.9 |
| miR-19a-3p | 319 | 1.69E-06 | 214 | -0.6 | 348 | 0.3 | 59 | -2.3 | 4950 | 4.1 |
| miR-19b-3p | 2446 | 2.24E-05 | 5496 | 1.0 | 4395 | 0.9 | 1165 | -1.1 | 10785 | 2.2 |
| miR-200c-3p | 29 | 5.42E-03 | 52 | 0.9 | 48 | 0.7 | 12 | -1.3 | 31 | 0.2 |
| miR-206 | 91 | 2.26E-03 | 21 | -2.6 | 69 | -0.5 | 122 | 0.2 | 6 | -4.7 |
| miR-20a/b-5p* | 3792 | 2.47E-05 | 8739 | 1.3 | 7025 | 0.9 | 238 | -8.2 | 27162 | 2.9 |
| miR-21-5p | 4237 | 1.20E-05 | 15814 | 1.7 | 25829 | 2.6 | 2092 | -1.1 | 12447 | 1.6 |
| miR-222-3p | 342 | 1.14E-08 | 235 | -0.6 | 1920 | 2.5 | 877 | 1.3 | 3495 | 3.4 |
| miR-223-3p | 449934 | 1.38E-06 | 83311 | -2.4 | 8281 | -6.2 | 5249 | -6.5 | 6075 | -6.1 |
| miR-22-3p | 367 | 1.36E-07 | 1342 | 1.8 | 300 | -0.3 | 12 | -5.6 | 6280 | 4.1 |
| miR-23a-3p | 8890 | 4.60E-07 | 13118 | 0.5 | 1262 | -2.8 | 161 | -5.9 | 164 | -5.8 |
| miR-23b-3p | 237 | 1.81E-06 | 686 | 1.6 | 67 | -1.8 | 5 | -5.7 | 125 | -0.9 |
| miR-24-3p | 3291 | 1.27E-08 | 8439 | 1.3 | 1839 | -0.9 | 225 | -3.9 | 269 | -3.6 |
| miR-25-3p | 10048 | 5.08E-08 | 7416 | -0.8 | 5165 | -1.0 | 1683 | -2.6 | 49547 | 2.3 |
| miR-26a-5p | 26292 | 1.36E-07 | 33364 | 0.3 | 31141 | 0.2 | 4259 | -2.6 | 3859 | -2.8 |
| miR-26b-5p | 1166 | 5.39E-09 | 604 | -1.0 | 3315 | 1.5 | 682 | -0.8 | 62081 | 5.8 |
| miR-296-5p | 64 | 1.68E-05 | 83 | 0.4 | 187 | 1.5 | 21 | -1.6 | 190 | 1.5 |
| miR-29a-3p | 9119 | 3.82E-07 | 2306 | -1.9 | 13388 | 0.7 | 2738 | -1.7 | 19 | -8.9 |
| miR-29b-3p | 516 | 9.90E-04 | 93 | -2.7 | 1461 | 1.5 | 150 | -2.2 | 353 | -0.5 |
| miR-29c-3p | 3783 | 1.36E-07 | 1583 | -1.3 | 8568 | 1.2 | 2033 | -1.0 | 1094 | -1.8 |
| miR-301a-3p | 285 | 5.90E-04 | 356 | 0.2 | 58 | -2.5 | 13 | -5.0 | 304 | 0.0 |
| miR-30a-5p | 432 | 7.26E-03 | 404 | -0.1 | 768 | 0.9 | 242 | -0.9 | 524 | 0.3 |
| miR-30b-5p | 2499 | 1.78E-07 | 4719 | 0.8 | 7904 | 1.6 | 1765 | -0.5 | 10143 | 2.0 |
| miR-30c-5p | 685 | 2.51E-04 | 339 | -1.1 | 356 | -1.0 | 57 | -3.7 | 751 | 0.2 |
| miR-30d-5p | 340 | 9.48E-10 | 333 | -0.1 | 534 | 0.6 | 126 | -1.4 | 3937 | 3.5 |
| miR-30e-5p | 1741 | 6.44E-06 | 147 | -3.6 | 2453 | 0.5 | 584 | -1.7 | 1924 | 0.2 |
| miR-32-5p | 136 | 2.91E-04 | 107 | -1.2 | 144 | 0.1 | 9 | -5.2 | 467 | 1.9 |
| miR-331-3p | 56 | 4.66E-05 | 170 | 1.6 | 68 | 0.3 | 18 | -2.2 | 361 | 2.7 |
| miR-335-5p | 125 | 8.38E-07 | 1552 | 3.6 | 131 | -0.1 | 22 | -3.2 | 49 | -1.4 |
| miR-340-5p | 2853 | 1.78E-07 | 1385 | -1.1 | 314 | -3.4 | 38 | -6.3 | 90 | -5.0 |
| miR-342-3p | 506 | 4.76E-09 | 259 | -1.0 | 21554 | 5.5 | 2614 | 2.4 | 428 | -0.2 |
| miR-34c-5p | 40 | 1.52E-06 | 32 | -0.3 | 33 | -1.0 | 4 | -4.0 | 412 | 3.4 |
| miR-361-3p | 80 | 5.90E-04 | 57 | -0.5 | 192 | 1.2 | 45 | -0.8 | 92 | 0.2 |
| miR-361-5p | 439 | 7.39E-07 | 463 | 0.1 | 235 | -0.9 | 22 | -4.4 | 40 | -3.4 |
| miR-363-3p | 13 | 3.98E-08 | 72 | 3.0 | 195 | 4.5 | 88 | 3.3 | 10674 | 10.3 |
| miR-374a-5p | 677 | 1.11E-07 | 1609 | 1.0 | 717 | 0.1 | 66 | -3.5 | 27360 | 5.4 |
| miR-374b-5p | 609 | 5.08E-08 | 975 | 0.7 | 478 | -0.4 | 43 | -3.9 | 4217 | 2.8 |
| miR-423-3p | 291 | 4.20E-05 | 542 | 0.8 | 334 | 0.2 | 52 | -2.5 | 116 | -1.5 |
| miR-423-5p | 834 | 4.77E-06 | 2010 | 1.2 | 596 | -0.5 | 234 | -1.9 | 2534 | 1.6 |
| miR-425-5p | 601 | 2.94E-07 | 373 | -0.7 | 179 | -1.7 | 51 | -3.6 | 2321 | 2.0 |
| miR-451a | 1067 | 4.15E-09 | 14217 | 3.6 | 121 | -2.9 | 116 | -3.1 | 1024619 | 10.3 |
| miR-454-3p | 388 | 3.82E-09 | 97 | -2.0 | 132 | -1.5 | 19 | -4.5 | 3788 | 3.3 |
| miR-484 | 427 | 6.48E-08 | 859 | 1.2 | 234 | -0.6 | 40 | -3.2 | 665 | 0.9 |
| miR-542-3p | 126 | 3.09E-05 | 44 | -2.2 | 16 | -3.1 | 28 | -2.4 | 5 | -5.1 |
| miR-548g-3p | 63 | 1.49E-03 | 70 | 1.2 | 22 | 0.1 | 43 | 1.2 | 156 | 3.1 |
| miR-660-5p | 51 | 1.11E-07 | 44 | -0.2 | 69 | 0.5 | 30 | -0.8 | 1256 | 4.7 |
| miR-720 | 7532 | 1.06E-06 | 3152 | -1.2 | 11026 | 0.3 | 3147 | -1.3 | 103 | -6.3 |
| miR-92a-3p | 4445 | 4.25E-06 | 19625 | 2.1 | 14499 | 1.7 | 4998 | 0.2 | 21592 | 2.3 |
| miR-92b-3p | 47 | 1.75E-05 | 156 | 1.9 | 89 | 1.0 | 24 | -1.0 | 2 | -4.9 |
| miR-93-5p | 478 | 1.65E-10 | 853 | 0.8 | 151 | -1.6 | 26 | -4.3 | 27493 | 5.9 |
| miR-96-5p | 210 | 4.66E-05 | 664 | 1.6 | 444 | 1.1 | 158 | -0.4 | 742 | 1.8 |
| miR-98 | 232 | 1.36E-07 | 1253 | 2.3 | 199 | -0.2 | 22 | -3.7 | 1226 | 2.4 |
| miR-99b-5p | 51 | 1.70E-04 | 303 | 2.4 | 34 | -0.9 | 7 | -4.3 | 18 | -1.6 |

* In a few cases, the probes used were not able to distinguish between similar sequences miRNAs.

*q*-value shows the statistical significance, adjusted for multiple testing, for the differential expression of granulocytes miRNA compared to all other sell types.

**Table S6 E. miRNAs DE in erythrocytes compared with all other cell types.**

|  | Counts |  | Counts | Fold(Log2) | Counts | Fold(Log2) | Counts | Fold(Log2) | Counts | Fold(Log2) |
| --- | --- | --- | --- | --- | --- | --- | --- | --- | --- | --- |
| Gene Name | **Erythro-**  **cytes** | ***q-*value** | **T-cells** | **T-cells *vs*. Erythr.** | **B-cells** | **B-cells *vs.* Erythr.** | **Granulo-**  **cytes** | **Gran. *vs.* Erythr.** | **Platelets** | **Platelets *vs*. Erythr.** |
| *let-7a-5p* | 105131 | 1.65E-10 | 8670 | -3.7 | 1796 | -5.9 | 4880 | -4.4 | 18937 | -2.5 |
| *let-7b-5p* | 56326 | 4.26E-10 | 1147 | -5.6 | 304 | -7.6 | 1020 | -5.8 | 1168 | -5.6 |
| *let-7c* | 4830 | 1.01E-07 | 132 | -5.3 | 13 | -9.1 | 88 | -5.9 | 87 | -5.9 |
| *let-7d-5p* | 5032 | 7.84E-05 | 3639 | -0.5 | 1157 | -2.1 | 3962 | -0.3 | 15925 | 1.4 |
| *let-7f-5p* | 8348 | 5.05E-07 | 5421 | -0.7 | 1133 | -2.9 | 2547 | -1.8 | 13395 | 0.6 |
| *let-7g-5p* | 73217 | 4.59E-07 | 55710 | -0.4 | 9844 | -2.9 | 9253 | -3.0 | 21228 | -2.0 |
| *let-7i-5p* | 18282 | 4.59E-07 | 3862 | -2.2 | 1421 | -3.7 | 1045 | -4.1 | 11471 | -0.7 |
| *miR-101-3p* | 226 | 1.78E-07 | 2469 | 3.5 | 458 | 0.9 | 1888 | 3.0 | 567 | 1.1 |
| *miR-103a-3p* | 142 | 6.66E-09 | 2758 | 4.3 | 573 | 2.1 | 4774 | 5.1 | 12976 | 6.5 |
| *miR-106a-5p/7-5p** | 31312 | 2.56E-11 | 252 | -7.0 | 50 | -9.3 | 171 | -7.5 | 365 | -6.4 |
| *miR-106b-5p* | 27992 | 5.56E-08 | 1870 | -3.9 | 538 | -5.8 | 3466 | -3.0 | 1607 | -4.2 |
| *miR-107* | 2704 | 8.51E-07 | 91 | -5.3 | 12 | -8.4 | 263 | -3.4 | 891 | -1.6 |
| *miR-10a-5p* | 11 | 2.61E-02 | 76 | 4.0 | 22 | 1.6 | 42 | 2.4 | 109 | 4.3 |
| *miR-125b-5p* | 182 | 1.14E-03 | 463 | 1.3 | 9 | -5.3 | 53 | -1.6 | 162 | 0.0 |
| *miR-1260a* | 127 | 3.05E-06 | 3816 | 5.0 | 2105 | 4.2 | 1222 | 3.2 | 185 | 0.6 |
| *miR-126-3p* | 32963 | 3.37E-08 | 450 | -6.2 | 85 | -8.7 | 148 | -7.9 | 85658 | 1.2 |
| *miR-128* | 123 | 2.73E-04 | 49 | -1.3 | 15 | -3.9 | 118 | -0.1 | 151 | 0.3 |
| *miR-1283* | 238 | 7.84E-05 | 72 | -1.8 | 124 | -1.1 | 87 | -1.5 | 6 | -5.4 |
| *miR-130b-3p* | 63 | 2.36E-04 | 26 | -1.4 | 4 | -4.7 | 39 | -0.7 | 153 | 1.2 |
| *miR-140-3p* | 142 | 6.86E-07 | 366 | 1.4 | 91 | -0.7 | 72 | -1.0 | 21 | -2.8 |
| *miR-140-5p* | 475 | 8.38E-07 | 429 | -0.1 | 61 | -3.0 | 860 | 0.8 | 253 | -1.0 |
| *miR-142-3p* | 35909 | 2.73E-04 | 119999 | 1.7 | 30119 | -0.5 | 112362 | 1.7 | 67408 | 0.7 |
| *miR-142-5p* | 37 | 2.12E-07 | 20928 | 9.3 | 3175 | 6.2 | 18903 | 9.2 | 2167 | 6.0 |
| *miR-146a-5p* | 76 | 3.50E-09 | 3614 | 5.6 | 325 | 2.0 | 31 | -1.3 | 3964 | 5.7 |
| *miR-146b-5p* | 96 | 4.56E-07 | 2186 | 4.5 | 34 | -3.2 | 18 | -2.4 | 40 | -1.3 |
| *miR-148a-3p* | 2035 | 2.01E-07 | 426 | -2.2 | 101 | -4.5 | 4168 | 1.0 | 1446 | -0.5 |
| *miR-148b-3p* | 1597 | 9.52E-07 | 507 | -1.7 | 138 | -3.6 | 1531 | -0.1 | 3993 | 1.3 |
| *miR-150-5p* | 90 | 2.56E-11 | 207293 | 11.2 | 28880 | 8.3 | 1090 | 3.5 | 38 | -1.4 |
| *miR-1537* | 47 | 4.59E-07 | 122 | 1.1 | 27 | -0.8 | 1687 | 5.2 | 99 | 0.5 |
| *miR-155-5p* | 97 | 3.55E-07 | 711 | 2.9 | 237 | 1.2 | 17 | -2.8 | 37 | -1.4 |
| *miR-15a-5p* | 84995 | 1.78E-07 | 4075 | -4.4 | 2009 | -5.4 | 16542 | -2.4 | 14117 | -2.8 |
| *miR-15b-5p* | 157204 | 3.82E-09 | 6766 | -4.5 | 1664 | -6.6 | 16374 | -3.3 | 20482 | -3.0 |
| *miR-16-5p* | 118502 | 5.64E-06 | 17938 | -2.7 | 9323 | -3.6 | 23256 | -2.3 | 20495 | -2.6 |
| *miR-181a-5p* | 1809 | 4.19E-04 | 3427 | 0.9 | 1017 | -0.8 | 2852 | 0.7 | 3159 | 0.4 |
| *miR-181c-5p* | 40 | 8.70E-05 | 47 | 0.2 | 2 | -4.7 | 57 | 0.6 | 40 | -0.1 |
| *miR-185-5p* | 8544 | 4.15E-09 | 95 | -6.5 | 9 | -10.0 | 175 | -5.6 | 592 | -3.9 |
| *miR-186-5p* | 1936 | 4.80E-08 | 971 | -1.0 | 133 | -3.9 | 512 | -1.9 | 189 | -3.4 |
| *miR-191-5p* | 5692 | 1.18E-06 | 1374 | -2.1 | 348 | -4.1 | 5705 | 0.0 | 8238 | 0.5 |
| *miR-194-5p* | 3063 | 9.48E-10 | 181 | -4.1 | 5 | -9.8 | 85 | -5.2 | 20 | -7.3 |
| *miR-197-3p* | 33 | 1.62E-04 | 41 | 0.1 | 4 | -3.9 | 572 | 3.8 | 164 | 2.2 |
| *miR-199a/b-3p** | 40 | 1.21E-07 | 72 | 0.8 | 10 | -3.0 | 596 | 3.9 | 14497 | 8.4 |
| *miR-19a-3p* | 4950 | 1.69E-06 | 348 | -3.8 | 59 | -6.4 | 319 | -4.1 | 214 | -4.7 |
| *miR-19b-3p* | 10785 | 2.24E-05 | 4395 | -1.3 | 1165 | -3.2 | 2446 | -2.2 | 5496 | -1.2 |
| *miR-200c-3p* | 31 | 5.42E-03 | 48 | 0.6 | 12 | -1.5 | 29 | -0.2 | 52 | 0.7 |
| *miR-206* | 6 | 2.26E-03 | 69 | 4.2 | 122 | 4.9 | 91 | 4.7 | 21 | 2.1 |
| *miR-20a/b-5p** | 27162 | 2.47E-05 | 7025 | -2.0 | 238 | -11.0 | 3792 | -2.9 | 8739 | -1.6 |
| *miR-21-5p* | 12447 | 1.20E-05 | 25829 | 1.0 | 2092 | -2.6 | 4237 | -1.6 | 15814 | 0.1 |
| *miR-222-3p* | 3495 | 1.14E-08 | 1920 | -0.9 | 877 | -2.1 | 342 | -3.4 | 235 | -3.9 |
| *miR-223-3p* | 6075 | 1.38E-06 | 8281 | -0.1 | 5249 | -0.4 | 449934 | 6.1 | 83311 | 3.7 |
| *miR-22-3p* | 6280 | 1.36E-07 | 300 | -4.4 | 12 | -9.7 | 367 | -4.1 | 1342 | -2.3 |
| *miR-23a-3p* | 164 | 4.60E-07 | 1262 | 3.0 | 161 | -0.1 | 8890 | 5.8 | 13118 | 6.2 |
| *miR-23b-3p* | 125 | 1.81E-06 | 67 | -0.9 | 5 | -4.8 | 237 | 0.9 | 686 | 2.5 |
| *miR-24-3p* | 269 | 1.27E-08 | 1839 | 2.8 | 225 | -0.2 | 3291 | 3.6 | 8439 | 5.0 |
| *miR-25-3p* | 49547 | 5.08E-08 | 5165 | -3.3 | 1683 | -4.9 | 10048 | -2.3 | 7416 | -3.1 |
| *miR-26a-5p* | 3859 | 1.36E-07 | 31141 | 3.0 | 4259 | 0.1 | 26292 | 2.8 | 33364 | 3.1 |
| *miR-26b-5p* | 62081 | 5.39E-09 | 3315 | -4.3 | 682 | -6.6 | 1166 | -5.8 | 604 | -6.8 |
| *miR-296-5p* | 190 | 1.68E-05 | 187 | 0.0 | 21 | -3.2 | 64 | -1.5 | 83 | -1.2 |
| *miR-29a-3p* | 19 | 3.82E-07 | 13388 | 9.6 | 2738 | 7.2 | 9119 | 8.9 | 2306 | 7.0 |
| *miR-29b-3p* | 353 | 9.90E-04 | 1461 | 1.9 | 150 | -1.7 | 516 | 0.5 | 93 | -2.2 |
| *miR-29c-3p* | 1094 | 1.36E-07 | 8568 | 3.0 | 2033 | 0.9 | 3783 | 1.8 | 1583 | 0.5 |
| *miR-301a-3p* | 304 | 5.90E-04 | 58 | -2.5 | 13 | -5.1 | 285 | 0.0 | 356 | 0.1 |
| *miR-30a-5p* | 524 | 7.26E-03 | 768 | 0.5 | 242 | -1.2 | 432 | -0.3 | 404 | -0.4 |
| *miR-30b-5p* | 10143 | 1.78E-07 | 7904 | -0.4 | 1765 | -2.5 | 2499 | -2.0 | 4719 | -1.2 |
| *miR-30c-5p* | 751 | 2.51E-04 | 356 | -1.2 | 57 | -3.9 | 685 | -0.2 | 339 | -1.2 |
| *miR-30d-5p* | 3937 | 9.48E-10 | 534 | -2.9 | 126 | -5.0 | 340 | -3.5 | 333 | -3.6 |
| *miR-30e-5p* | 1924 | 6.44E-06 | 2453 | 0.4 | 584 | -1.9 | 1741 | -0.2 | 147 | -3.8 |
| *miR-32-5p* | 467 | 2.91E-04 | 144 | -1.8 | 9 | -7.0 | 136 | -1.9 | 107 | -3.0 |
| *miR-331-3p* | 361 | 4.66E-05 | 68 | -2.5 | 18 | -5.0 | 56 | -2.7 | 170 | -1.1 |
| *miR-335-5p* | 49 | 8.38E-07 | 131 | 1.2 | 22 | -1.8 | 125 | 1.4 | 1552 | 4.9 |
| *miR-340-5p* | 90 | 1.78E-07 | 314 | 1.6 | 38 | -1.3 | 2853 | 5.0 | 1385 | 3.9 |
| *miR-342-3p* | 428 | 4.76E-09 | 21554 | 5.6 | 2614 | 2.6 | 506 | 0.2 | 259 | -0.8 |
| *miR-34c-5p* | 412 | 1.52E-06 | 33 | -4.4 | 4 | -7.4 | 40 | -3.4 | 32 | -3.7 |
| *miR-361-3p* | 92 | 5.90E-04 | 192 | 1.0 | 45 | -1.0 | 80 | -0.2 | 57 | -0.7 |
| *miR-361-5p* | 40 | 7.39E-07 | 235 | 2.5 | 22 | -1.0 | 439 | 3.4 | 463 | 3.5 |
| *miR-363-3p* | 10674 | 3.98E-08 | 195 | -5.8 | 88 | -7.0 | 13 | -10.3 | 72 | -7.3 |
| *miR-374a-5p* | 27360 | 1.11E-07 | 717 | -5.3 | 66 | -8.8 | 677 | -5.4 | 1609 | -4.4 |
| *miR-374b-5p* | 4217 | 5.08E-08 | 478 | -3.2 | 43 | -6.7 | 609 | -2.8 | 975 | -2.1 |
| *miR-423-3p* | 116 | 4.20E-05 | 334 | 1.6 | 52 | -1.1 | 291 | 1.5 | 542 | 2.3 |
| *miR-423-5p* | 2534 | 4.77E-06 | 596 | -2.1 | 234 | -3.5 | 834 | -1.6 | 2010 | -0.4 |
| *miR-425-5p* | 2321 | 2.94E-07 | 179 | -3.7 | 51 | -5.5 | 601 | -2.0 | 373 | -2.6 |
| *miR-451a* | 1024619 | 4.15E-09 | 121 | -13.1 | 116 | -13.4 | 1067 | -10.3 | 14217 | -6.7 |
| *miR-454-3p* | 3788 | 3.82E-09 | 132 | -4.8 | 19 | -7.8 | 388 | -3.3 | 97 | -5.3 |
| *miR-484* | 665 | 6.48E-08 | 234 | -1.5 | 40 | -4.1 | 427 | -0.9 | 859 | 0.4 |
| *miR-542-3p* | 5 | 3.09E-05 | 16 | 2.1 | 28 | 2.8 | 126 | 5.1 | 44 | 3.0 |
| *miR-548g-3p* | 156 | 1.49E-03 | 22 | -3.0 | 43 | -1.9 | 63 | -3.1 | 70 | -1.9 |
| *miR-660-5p* | 1256 | 1.11E-07 | 69 | -4.2 | 30 | -5.5 | 51 | -4.7 | 44 | -4.9 |
| *miR-720* | 103 | 1.06E-06 | 11026 | 6.5 | 3147 | 5.0 | 7532 | 6.3 | 3152 | 5.0 |
| *miR-92a-3p* | 21592 | 4.25E-06 | 14499 | -0.6 | 4998 | -2.1 | 4445 | -2.3 | 19625 | -0.2 |
| *miR-92b-3p* | 2 | 1.75E-05 | 89 | 5.9 | 24 | 3.8 | 47 | 4.9 | 156 | 6.7 |
| *miR-93-5p* | 27493 | 1.65E-10 | 151 | -7.5 | 26 | -10.1 | 478 | -5.9 | 853 | -5.1 |
| *miR-96-5p* | 742 | 4.66E-05 | 444 | -0.7 | 158 | -2.3 | 210 | -1.8 | 664 | -0.2 |
| *miR-98* | 1226 | 1.36E-07 | 199 | -2.6 | 22 | -6.1 | 232 | -2.4 | 1253 | -0.1 |
| *miR-99b-5p* | 18 | 1.70E-04 | 34 | 0.7 | 7 | -2.6 | 51 | 1.6 | 303 | 4.1 |

* In a few cases, the probes used were not able to distinguish between similar sequences miRNAs.

*q*-value shows the statistical significance, adjusted for multiple testing, for the differential expression of erythrocytes miRNA compared to all other sell types.
