# Supplementary material for: MicroRNA Expression Differences in Human Hematopoietic Cell Lineages Enable Regulated Transgene Expression
Source: PLoS One. 2014 Jul 16;9(7):e102259. doi: 10.1371/journal.pone.0102259 (PMC4100820; doi:10.1371/journal.pone.0102259)
Supplement: Table S7 — Selectively reduced miRNAs amongst abundantly expressed miRNAs. (DOCX) [file pone.0102259.s010.docx]

**Table S7. Selectively reduced miRNAs amongst abundantly expressed miRNAs.**

|  | **Erythrocyte** | **Platelet** | **Granulocyte** | **T-cell** | **B-cell** |
| --- | --- | --- | --- | --- | --- |
| *miR-142-5p* | 37 | 2,167 | 18,903 | 20,928 | 3,175 |
| *miR-29a-3p* | 19 | 2,306 | 9,119 | 13,388 | 2,738 |
|  |  |  |  |  |  |
| *miR-150-5p* | 90 | 38 | 1,090 | 207,293 | 28,880 |
|  |  |  |  |  |  |
| *miR-93-5p* | 27,493 | 853 | 478 | 151 | 26 |
