# Supplementary material for: MicroRNA Expression Differences in Human Hematopoietic Cell Lineages Enable Regulated Transgene Expression
Source: PLoS One. 2014 Jul 16;9(7):e102259. doi: 10.1371/journal.pone.0102259 (PMC4100820; doi:10.1371/journal.pone.0102259)
Supplement: Table S8 — miR-30c-5p validation. (DOCX) [file pone.0102259.s011.docx]

**Table S8. *miR-30c-5p* validation**.

|  | *miR-30c-5p* | *RNU6B* |
| --- | --- | --- |
| Average of Ct | 17.55 | 29.83 |
| Average of SD | 0.65 | 0.94 |

Ct: Cycle threshold; SD: Standard deviation.

We compared expression levels and variability of *miR-30c-5p* and *RNU6B* among 5 subjects and each of 5 cells types by qRT-PCR using the same amount of starting total RNA. Compared to *miR-30c-5p, RNU6B* is expressed at a much lower average level (as reflected by higher cycle threshold [Ct]) and displays greater variability (higher SD).
